# Developing a web-based platform for *Eragrostis tef* resource management and analysis

**DOI:** 10.1093/database/baag051

**Published:** 2026-08-25

**Authors:** Badasa Firdisa Fufa, Adugna Abdi Woldesemayat, Abiy Zegeye

**Affiliations:** Institute of Biotechnology, Addis Ababa University, P.O. Box: 1176, Addis Ababa, Ethiopia; Departments of Biotechnology, Addis Ababa Science and Technology University, P.O. Box: 16417, Addis Ababa, Ethiopia; Departments of Biotechnology, Addis Ababa Science and Technology University, P.O. Box: 16417, Addis Ababa, Ethiopia; Institute of Biotechnology, Addis Ababa University, P.O. Box: 1176, Addis Ababa, Ethiopia

## Abstract

Developing a web-based platform is beneficial for data accessibility, centralization, security, management, and scalability. *Eragrostis tef* is an indigenous cereal crop in Ethiopia that currently lacks specialized or unified web-based platforms compared with major crops. This gap hinders genomic research, crop improvement programs, and the efficient utilization of tef genetic resources. Consequently, challenges include data fragmentation and the absence of a centralized database for accessing and managing tef-related data. In this study, a centralized platform called the Tef Database (TefDB) was developed. The platform integrates 77 795 tef-specific biological records, including 1 372 literature entries, 6 144 genome-related records, 66 455 gene models, 3 447 protein sequences, and 377 chemical/pathway entries. Advanced features were included along with the platform’s interactive, visualization, responsive, searchable and filterable, downloadable, and analysis tools. The platform was built using modern web technologies: React.js for the frontend, Django and Python for backend processing, and PostgreSQL for data management. The Design Science Research (DSR) methodology guided development, incorporating iterative input from potential users. The platform’s usability, performance, and functionality were validated through testing. TefDB offers bioinformatics analysis through natively embedded tools (BLAST and JBrowse 2) and externally linked tools (Primer3Plus and Clustal Omega) that open in new browser tabs. This integration reduces workflow fragmentation, allowing users to move seamlessly from data retrieval to analysis without exporting datasets to external platforms. Future work should focus on platform upgrades, data updates and integration, integration of local tef variety data (including SNPs and VCFs), advanced analytical modules, and collaborative user features.

## Introduction

*Eragrostis tef* is a climate-resilient cereal and staple crop in the Horn of Africa, cultivated by millions of smallholder farmers [1]. It is rich in complex carbohydrates, dietary fiber, and essential minerals such as calcium, iron, and zinc, as well as an amino acid (i.e., high lysine content) often lacking in other grains [2]. Additionally, tef flour is used to make the traditional flatbread injera as well as pancakes, porridge, and alcoholic beverages such as *tella*. The demand for tef product is also increasing globally due to its health benefits as a gluten-free grain [3]. However, tef productivity is low compared with other cereals [4]. In Ethiopia, tef is a vital source of income for millions of smallholder farmers, with the grain serving as a staple food for over 70 million people [1]. Despite these advantages, tef potential remains largely untapped outside its region of origin due to limited research funds and the absence of a specialized, species-specific database. Existing resources such as NCBI, EnsemblPlants, plantTFDB, and UniProt include tef data but are not tailored to tef-specific research needs. This fragmented landscape creates inefficiencies for researchers who must navigate multiple platforms to access and analyze tef-related data.

This study addresses these gaps by developing a comprehensive web-based platform for tef resource management and analysis. The platform integrates various types of biological data related to tef, such as genomic, phenotypic, proteomic, and literature, and creates a centralized repository that can be accessed and analyzed by users such as researchers, bioinformaticians, students, and agricultural stakeholders alike. The integrated bioinformatic tools enable users to analyze complex datasets and generate insights about tef. The platform helps bridge the knowledge gap, promotes the crop’s global recognition, and encourages product innovation [5].

Moreover, with future updating, upgrading, and expansion, the platform will serve as a space for collaboration among international and Ethiopian researchers, fostering knowledge exchange and increasing the importance of tef an important cereal crop. By providing an interactive environment, the platform will enable researchers and stakeholders to actively deposit, update, manage, and share tef-related data. It will facilitate continuous data exchange, user contributions, and collaborative engagement among platform users, thereby fostering dynamic global interaction aimed at optimizing tef genetic resources and improving production traits.

## Methodology

This research used the design science research (DSR) approach [6, 7] to develop and evaluate an interactive web platform called **TefDB** (**Tef D**ata**B**ase), designed for the management and analysis of *Eragrostis tef* biological resources. DSR is an appropriate methodological framework for this study because the aim is to create, construct, and rigorously assess a technological artifact that addresses a clearly defined problem—namely, the data fragmentation across repositories and lack of specialized and unified databases for *E. tef* biological data resources.

The DSR approach was implemented through three interrelated cycles [8]: relevance cycle, rigor cycle, and design cycle.

The relevance cycle linked the research to a real-world problem or challenges in the data management and analysis, including data fragmentation and no specialized databases for *E. tef* by ensuring that TefDB meets a user’s need. The user requirements were collected through a structured survey from researchers, bioinformaticians, biotechnologists, educators or teachers, and students who are considered potential beneficiaries of the platform. The feedback garnered from potential users served to guide the TefDB design system and ensure the developed artifacts addressed the identified requirements regarding *E. tef* biological resources.

The rigor cycle was developed based on the published existing scientific knowledge, methods, technologies, and theories. The rigor cycle was grounded in existing scientific literature on biological databases, database design principles (such as usability, data integrity, entity integrity, and performance), and FAIR (Findable, Accessible, Interoperable, Reusable) standards.

Moreover, some specialized cereal crop databases, such as MaizeGDB (https://www.maizegdb.org/), SorghumBase (https://www.sorghumbase.org/), and Oryzabase (https://shigen.nig.ac.jp/rice/oryzabase/locale/change?lang=en), were reviewed. This insight guided the database schema, data modeling, the appearance of the platform, and data integrity, ensuring that the system, or TefDB, was methodologically aligned with the existing standards.

The design cycle involved the iterative design, implementation, demonstration, and evaluation of the TefDB. Biological datasets (nucleotide, genome, protein sequences, literature, *etc*.) for *Eragrostis tef* were integrated using automated API-based scripts and downloaded from public repositories: NCBI GenBank and Nucleotide databases, Ensembl Plants, and UniProt. This automated workflow data extraction ensured consistency and efficiency in data integration. Through these cycles, the TefDB was developed with *E. tef* resources and bioinformatic tools integrated as a fully functional, reliable, and user-interactive database that addresses the identified problems.

### Development workflow

**Figure 1:**
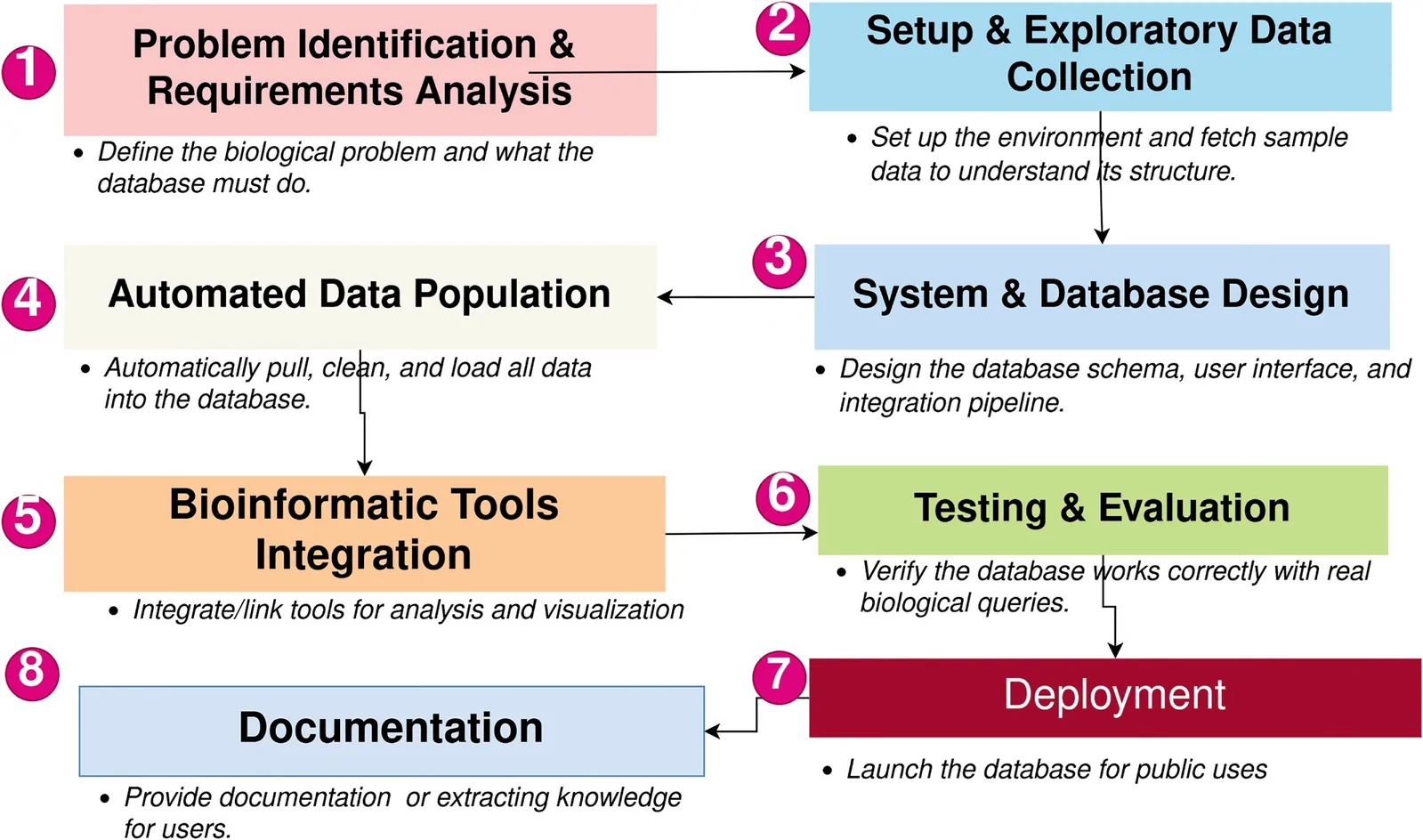
Workflow diagram of the study showing the sequential phases from problem identification to deployment and documentation, following the Design Science Research (DSR) methodology.

The development used a multi-phase approach supported by design science research (DSR) principles. Fig. 1 shows the comprehensive high-level research workflow of this study. The process involved sequential but iterative stages, starting with identifying the problem and analyzing requirements, then moving on to development setup environment, designing and developing artifacts (including database design, front-end, and back-end design), data population, bioinformatic tools integration, evaluation, deployment, and extracting knowledge or documentation.

### Phase 1: Problem identification and requirements analysis

In this phase the problems of data fragmentation and lack of a centralized platform for tef, and requirements to be solved and the system needed to do were identified. The analysis was conducted through the structured questionnaires involving researchers, students, teachers, and bioinformaticians; document analysis; and observation of existing systems. The requirements are defined as functional and non-functional requirements (see Table 2 & Table 3), which guided the system design and implementation.

### Phase 2: Setup development environment

The initial setup focused on creating a stable and reproducible environment for all components of TefDB. This included the operating system, code editors, version control systems, and database management. The technologies used for this setup environment development were summarized in Table 1.

**Table 1:** Development environment setup tools used for TefDB, including operating system (Ubuntu 22.04), IDE (VS Code), version control system (Git), and database management system (PostgreSQL).

Item	Tools	Purpose	Description
1	Ubuntu 22.04	Operating System	Provides a stable, secure, and open-source environment for development and deployment, compatible with bioinformatics tools.
2	VS Code	IDE/Code Editor	Main integrated environment for coding, debugging, and project management, with support for multiple languages and extensions.
3	Git	Version Control	Tracks changes in source code, supports version history, and allows rollback to previous states.
4	PostgreSQL	Database Management	Relational database to store and manage structured bioinformatics data.

### Phase 3: System design and implementation

#### Frontend development environment

Frontend development encompasses all aspects of user interface creation, from semantic web-page markup to complex dynamic user interactions. The core technologies behind frontend development included were summarized in Table 2 below with their purpose. In this study, Node.js was used exclusively as a front-end build tool and package manager (via npm) and not as a backend execution framework.

**Table 2:** Frontend development tools and frameworks used in TefDB, including HTML, CSS, JavaScript, Node.js, and React.js, with their respective purposes.

Item	Tools	Purpose	Description
1	HTML	Markup Language	Structures web pages and defines the content layout.
2	CSS	Styling	Styles HTML elements and ensures responsive and visually appealing interfaces.
3	JavaScript	Programming Language	Adds interactivity and dynamic behavior to web pages.
4	Node.js	Runtime Environment	Executes JavaScript outside the browser and supports frontend build processes.
5	React.js	Frontend Framework	Implements a component-based architecture, facilitating modular UI development, reusability, and efficient state management.

#### Backend development environment

Backend development is responsible for server-side logic, data processing, database management, and the execution of business processes. This backend development was designed by using various tools (see Table 3).

**Table 3:** Backend development and testing tools used in TefDB, including Python, Django, and Django REST Framework.

Item	Tools	Purpose	Description
1	Python	Programming Language	Implements server-side logic and data processing for backend functionality.
2	Django	Backend Framework	Provides a secure, scalable framework for web application development and RESTful API support.
3	Django REST Framework (DRF)	API Development	Exposes backend services to the frontend through RESTful APIs.

### Phase 4: Data collection and populate methods

During this research, two main categories of data were collected: biological data and user-centered data.

#### Biological data sources

Biological data for *Eragrostis tef* were collected from NCBI GenBank and Nucleotide databases, UniProt, and EnsemblPlants using automated scripts. From NCBI, the Entrez Programming Utilities (E-utilities) via Biopython retrieved 3,683 nucleotide sequences, 1,162 SRA runs, 23 BioProjects, 1,273 BioSamples, 203 PubMed records, and 1 taxonomy entry. FTP downloads provided GenBank flat files and 1,167 PubMed Central articles. From UniProt, the RESTful API returned 220 protein records (10 Swiss-Prot reviewed, 210 TrEMBL unreviewed). From EnsemblPlants, FTP download yielded one complete nuclear genome and 66,287 gene models. Metadata—including source repository, version number, release date, and access date—was stored in a PostgreSQL metadata table for reproducibility. All datasets were parsed using Python (Biopython, pandas, and gffutils) and validated with integrity checks (record count, format validation, and duplicate detection).

#### User-centered data

To ensure that the developed system meets user needs and expectations, user-centered data have been collected using several methods.

**Questionnaires:** Questionnaires were distributed to potential users (such as researchers, students, and teachers) to gather information on requirements analysis, system features, expected usability, and challenges faced on existing platforms.

**Observation of existing databases**: A systematic review and comparative assessment of established bio-databases and Cereal data platforms were conducted to examine their analytical tools, data integration strategies, and overall platform design. The databases included in this assessment are summarized in Table 4. The insights gained from this evaluation informed the structural framework, functionality, and feature selection implemented in TefDB.

**Table 4:** Cereal databases reviewed during the development of TefDB, including MaizeGDB, Wheat URGI, Sorghumbase, and Oryzabase, with their integrated tools and data types.

Item	Database	Tool integrated	Data type available
1	MaizeGDB	BLAST, SNPversity 2.0, Bin viewer, Genome viewer (GCV), GenomeQC, PAST, *etc*.	Genome, Protein structure, SNPs/Traits Alleles/Polymorphisim, Molecular marker, Expression, Loci + QTL, Maps, Gene Product, *etc*.
2	Wheat URGI	BLAST, JBrowse, Wheat Synteny View, Sirus Quality, Intermine, *etc*.	Assemblies, Annotations, Vaiations, Expression, Publications, Physical & genetic maps, Chromatin Accessibility, *etc*.
3	Sorghumbase	BLAST, Gene Search, and Genome Browser	Genome Assembly, Gene expession, Pathways, Genetic variation, *etc*.
4	Oryzabase	Seq. Cutter, Map Tool, Rice ID Checker, SeqAnalysis, *etc*.	Genome, Organs & Stages, Map, *etc*.

**Document Review**: Relevant documents such as literature, existing database documentation, and biological bioinformatics were reviewed to inform the system design and data structuring in the TefDB development [9–11].

### Phase 5: Bioinformatic tools integration

The integration process for BLAST and JBrowse 2 involved local installation, whereas Primer3Plus and Clustal Omega were integrated via external web links, which redirect users to the respective service websites. The integration was carried out using command-line execution, application programming interfaces (APIs), or embedded scripts, depending on the technical compatibility and accessibility of each tool.

The integration process followed an iterative procedure, in which each tool was repeatedly tested, evaluated, and refined based on system performance criteria and user feedback. This ensured proper functionality, reliability, and consistency within the system environment. The outputs generated from these tools were organized and presented through visualization components, including graphs, tables, and genome feature views, to support clear interpretation of results.

### Phase 6: Testing and evaluation

In this phase, the evaluation was performed to assess the effectiveness, usability, accessibility, and reliability of the system developed. Many evaluation techniques were used, including functional testing and performance testing by using the Postman application to collect metrics such as throughput, response time, status, and error rates [12,13,14] and Selenium tools for UI automating testing. The user-based evaluation through questionnaires was also carried out to validate the developed system. The evaluation results were analyzed using both qualitative and quantitative methods, and findings were used to iteratively refine the system.

### Phase 7: Deployment and documentation

The TefDB system was deployed in a production environment to enable real-world accessibility, usability testing, and system evaluation. The deployed system is accessible via its public IP address (http://197.156.76.122/), allowing users to interact with the platform over the internet, and the source code is available on GitHub (https://github.com/bioinfo33/MyProject).

A Linux-based server environment was configured to host the application. Nginx was used as the web server to manage HTTP requests and reverse proxy services, while Gunicorn was employed as the application server to execute the backend system and handle concurrent user requests. This deployment architecture supports efficient request handling, scalability, and system reliability.

The deployment process involved preparing a production-ready version of the application, transferring system files to the server, configuring the virtual environment and required dependencies, establishing database connections, and integrating Nginx with Gunicorn for proper request routing. Basic security configurations, including firewall setup and controlled access mechanisms, were also implemented.

In addition, system documentation was developed to support usability and user adoption. This included a user manual and API documentation (http://197.156.76.122/help), Frequently Asked Questions (FAQs), and integrated feedback and contact mechanisms. These resources facilitate user interaction and support continuous system improvement.

## Design and development

This section describes the design and development of the web-based platform, TefDB, for the management and analysis of *Eragrostis tef* resources. The design artifacts, system architecture, database structure, backend and frontend implementation, and bioinformatics tools integration were developed following an iterative, modular approach based on the requirements identified.

### Requirement analysis and classification

The gathered requirements were classified into functional and non-functional requirements. Functional requirements included data visualization, search, retrieval, analysis and integration with bioinformatics tools. Non-functional requirements addressed system performance, scalability, security, usability, and maintainability.

#### Functional requirements

Functional requirements define the expected service and system behavior from the user’s and administrator’s point of view. These requirements came from different users, and relate to access, exploration, analysis, and interaction of data. They specify what the system should allow users to do, such as search data, visualize biological data and information, and export results. Table 5 summarizes the functional requirements that should be included.

**Table 5:** Functional requirements of TefDB, listing system capabilities including data management, authentication, searching, visualization, downloading, feedback, and activity tracking.

Id	Description	Status
FR01	The system shall allow administrators to insert or add, update, delete and manage resource data stored in the PostgreSQL database.	Implemented
FR02	The system shall provide secure authentication and authorization, allowing users to sign up, log in, and manage their profiles.	Implemented
FR03	The system shall allow users to browse, search, analyse, and filter *Eragrostis tef* datasets, including genes, nucleotides, proteins, literature, *etc*.	Implemented
FR04	The system shall provide interactive data visualization dashboards for exploring datasets.	Implemented
FR05	The system shall enable users to copy, download, and export data reports in standard formats (such as JSON, XML, genbank, FASTA, and genpept).	Implemented
FR06	The system shall provide a feedback and contact module, allowing users to submit questions, report issues, and request new features.	Implemented
FR07	The system shall record and display recent activities, including data updates, delete, create, and user interactions, to support transparency and traceability.	Implemented

#### Non-functional requirements

Non-functional requirements specify criteria that evaluate how a system performs a function, rather than the function itself. These include usability, scalability, performance, security, maintainability, and FAIR data (Findable, Accessible, Interoperable, Reusable). Table 6 summarizes the non-functional requirements expected in the services and systems.

**Table 6:** Non-functional requirements of TefDB, including usability, scalability, performance, security, and FAIR data compliance.

Id	Description	Status
NFR01	The system shall be web-based and accessible on web browsers and devices.	Implemented
NFR02	The system shall be scalable, allowing integration of additional datasets and analytical modules without degrading performance.	Implemented
NFR03	The system shall ensure efficient data storage and retrieval through a relational database designed.	Implemented
NFR04	The system shall implement security measures, including authentication, authorization, and encrypted data transmission via HTTPS.	Partially implemented (JWT and CSRF implemented in code; HTTPS pending deployment)
NFR05	The system shall provide an intuitive and user-friendly interface with consistent navigation and accessibility.	Implemented
NFR06	The system shall comply with FAIR data principles (Findable, Accessible, Interoperable, Reusable) to support effective biological data management.	Implemented

### System architecture design

**Figure 2:**
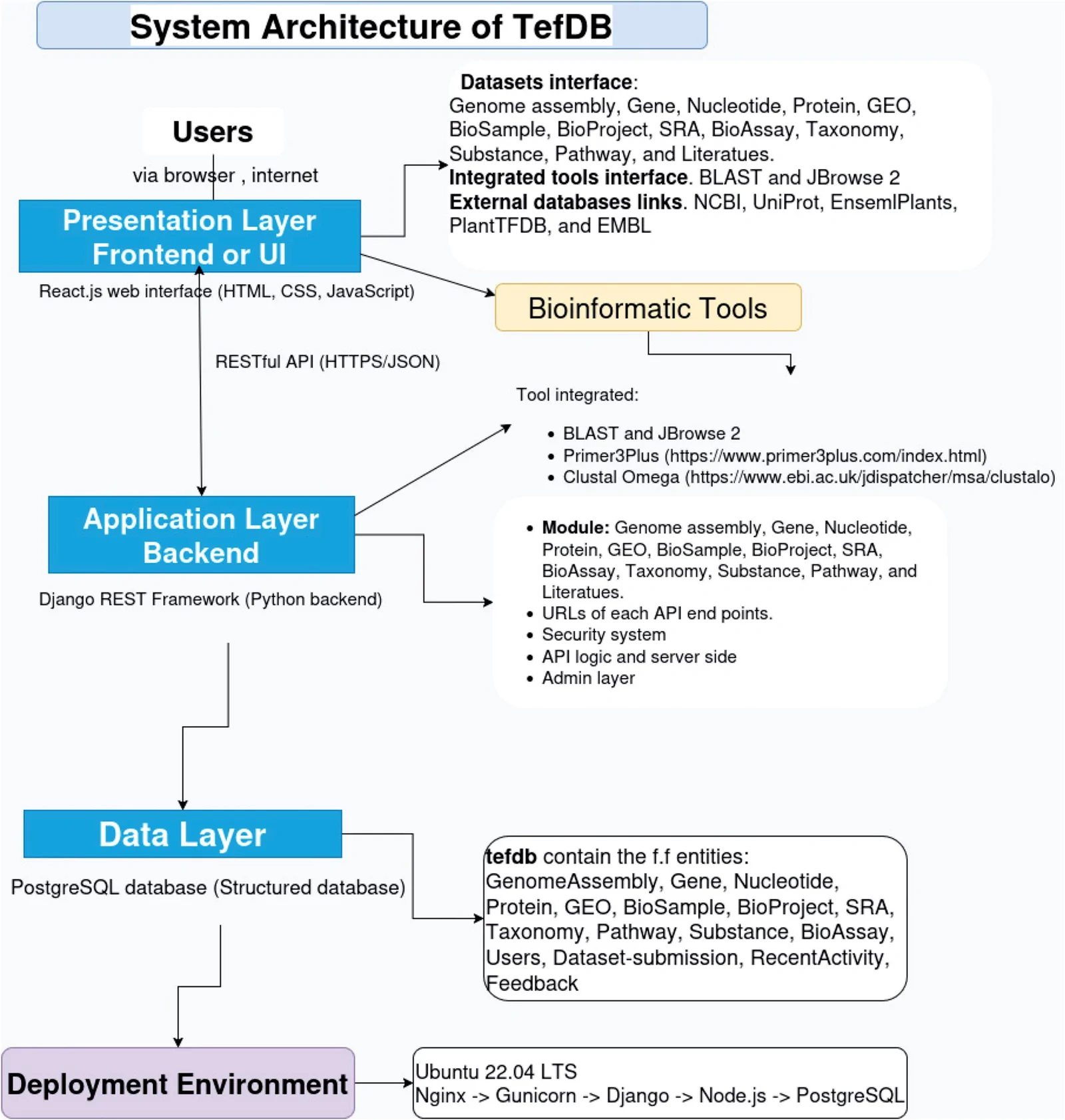
System architecture of TefDB illustrating the three-tier design: presentation layer (React.js frontend), application layer (Django backend with RESTful APIs), and data layer (PostgreSQL database).

TefDB was designed with a modular, multi-tier architecture to separate concerns, maintainability, and scalability. The architecture consists of: presentation layer, application layer, and data layer. The high-level system architecture and the interaction between components are shown on Fig. 2.

This architecture allows system components to be developed and scaled independently, while still allowing integration with bioinformatic tools.

**Presentation Layer** (Frontend): The user interface of the web-based platform. The frontend is responsible for the visual aspects of the web-based platform, such as the design of the user interface, the layout of the screens, and the navigation. An interactive interface with search bars, filterable genome viewers, and downloads is powered by ReactJS and Tailwind CSS.

**Application Layer** (Backend): The application layer of the system is implemented using the Django framework. This layer is responsible for request processing, coordinating data integration, and exposing application programming interfaces (APIs) to the client-side components.

Specifically, the backend manages communication with external biological data services, including the National Center for Biotechnology Information (NCBI) Entrez utilities, such as E-Search, E-Fetch, and E-Summary. Through these services, the system retrieves relevant biological datasets, parses and transforms the returned data into structured formats, and integrates them into the application workflow.

The application layer also incorporates an admin layer, which provides administrative control and system governance. This layer facilitates role-based access management, user and permission administration, dataset moderation, system configuration, and logging of administrative activities. The administrative interface is implemented using Django’s built-in admin module, which ensures that only authorized personnel with appropriate privileges can access sensitive functions and configuration settings.

To maintain security, all public and administrative endpoints are protected in the backend code using JSON Web Token (JWT)-based authentication, and Cross-Site Request Forgery (CSRF) protection is applied where appropriate. However, the current production deployment uses HTTP rather than HTTPS (as shown in Fig. 13). Without TLS encryption, JWT tokens are transmitted in plaintext, which is a security vulnerability. This is a recognized limitation of the current prototype deployment (see Limitations section). Future work will implement SSL/TLS certificates to enable full HTTPS, at which point the JWT and CSRF mechanisms will provide complete security.

**Data Layer** (Database): Biological data and information were stored in PostgreSQL in a modular schema with each entity (Gene, Protein, Nucleotide, PubMed, etc.) modeled as a table with primary and foreign keys, with indexes for performance optimization.

In general, the architecture of TefDB is based on a layered web-based architecture with a modern frontend, a strong backend framework, embedded bioinformatic tools, and a relational database system; a React-based frontend allows interactive access to tef-specific datasets and analysis tools, the Django backend arranges data ingestion, processing, and bioinformatic workflows, and PostgreSQL provides reliable and scalable data storage, and integration with external biological databases ensures comprehensive annotation and cross-referencing, leading to a unified and extensible platform for *Eragrostis tef* research.

### Database design and data integration

A relational database model was designed to manage various *E. tef* resources efficiently by the PostgreSQL database. The database schema was developed using Entity-Relationship (ER) modeling, representing core biological entities, attributes, and their relationships.

### Data integration from public repositories

Biological data and information were obtained from trusted public bioinformatics repositories, including NCBI, UniProt, and EnsemblPlants, using automated API-based scripts. Since these repositories provide standardized and curated datasets, no additional biological preprocessing or manual curation was performed. The retrieved data were mapped to the designed database schema and inserted directly into the TefDB designed by PostgreSQL database. Basic integrity checks were conducted to ensure successful data retrieval and consistency during insertion.

#### Conceptual data model

The Entity-Relationship Model (ER Model) is a conceptual model for designing a database. This model represents the logical structure of a database, including entities, attributes, and relationships between them.

**Entity:** An object that is stored as data. E.g., protein, Nucleotide, Genome assembly, PubMed, *etc*.

**Attribute:** Properties that describe an entity. E.g., protein_id, protein_name, scientific_name, *etc*.

**Relationship:** A connection between entities. E.g., each genome assembly belongs to exactly one organism; nucleotide entries can be mapped to or associated with genome assemblies.

**Cardinality:** Show whether the relationship is 1-to-1, 1-to-many, or many-to-many.

**Primary key** (PK): A candidate key chosen by the database designer to uniquely identify the entity set.

**Foreign key** (FK): Identifies the relationship between entities.

Key entities include Gene, Protein, Nucleotide, Genome Assembly, Taxonomy, BioProject, BioSample, SRA, Bio-assay, Pathway, Substance, GEO, Literature, User, Feedback, Dataset submissions, and Recent Activity.

**Figure 3:**
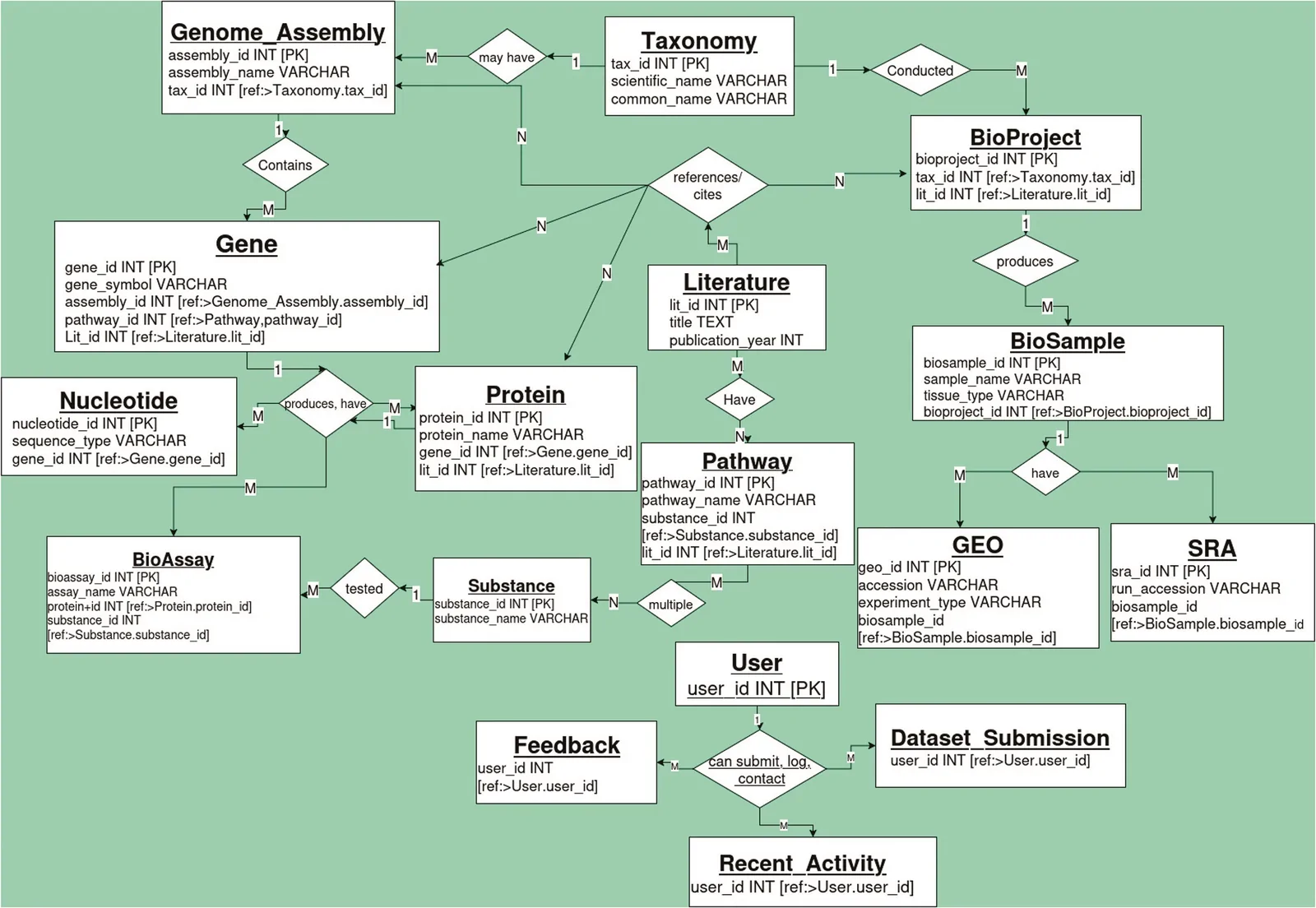
Entity–Relationship (ER) diagram of the TefDB database showing 17 entities, including Gene, Protein, Nucleotide, Genome Assembly, Taxonomy, BioProject, BioSample, SRA, GEO, Bio-assay, Pathway, Substance, Literature, User, Feedback, Recent Activity, and Dataset Submission, along with their relationships and cardinalities.

Relationships between entities were defined using primary and foreign keys to ensure referential integrity. Therefore, the ER diagram of TefDB was designed as below by using the web-based tool draw. io (https://www.drawio.com/) (see Figure 3):

**Components**:

**Rectangles**: Represent entities (tables).

**Diamonds**: Represent relationships between entities.

**Lines/Connectors**: Show links between entities and attributes, often with cardinality (e.g., one-to-many, many-to-many).

**Key to Cardinality Notations**:

**1: N** = One-to-Many (One instance of Entity1 relates to many instances of Entity2)

**N: M** = Many-to-Many (Many instances of Entity1 relate to many instance of Entity2)

An entity is a definable object that can have data stored about it. As shown in Fig. 2 shown there are 17 entities in this database.

**Taxonomy**: Represents the species Eragrostis *tef*.

**Genome Assembly**: The assembled DNA sequence of the whole genome.

**Gene**: A DNA region that encodes a functional product.

**Protein**: The translated product of a gene.

**Nucleotide**: Stores the DNA or RNA sequences.

**BioProject**: High-level project describing sequencing, experimental design.

**BioSample**: Biological material collected for sequencing or experiments.

**SRA (Sequence Read Archive)**: Raw sequencing reads.

**GEO (Gene Expression Omnibus)**: Expression data (RNA-seq/microarray).

**Bioassay:** Experimental test measuring biological activity.

**Pathway**: A biological process involving multiple proteins.

**Substance**: Chemical compound.

**Literature (PubMed, PMC, Bookshelf)**: Published scientific articles.

**Users:** Stores the user’s information.

**Feedback**: Stores the comment or question from users.

**Recent Activity**: Stores the action.

**Dataset submission**: Stores the new data submitted.

### Backend design and implementation

The backend component allows server-side logic, secure data access, processing, and communication between system components.

#### Backend architecture

The backend was designed using the Django framework, adhering to the Model–View–Template (MVT) architectural pattern, which promotes separation between data models, request handling, and response rendering. The core backend components are as follows:

**Models**: Define database schema, structure, and relationships.

**Views**: Handle request processing and business logic.

**Serializers**: Convert model instances to structured data formats.

**URLs**: Map API endpoints to backend services.

**Admin Interface**: Enables dataset and user management.

**Programming languages and frameworks:** Server-side development was performed using the programming language Python and Django (a high-level Python framework). These tools enable the implementation of complex business logic, high scalability, and support for modern web environments.

**API and integration solutions:** Standardized interfaces, implemented through architectural styles, REST, were used for effective communication between frontend and backend systems. REST provides a straightforward and easily understandable data exchange format.

**Database management systems (DBMS):** The choice of DBMS—whether relational (MySQL, PostgreSQL) or non-relational (MongoDB)—directly influences data storage structures and management strategies. Relational DBMS are well-suited for structured data, whereas NoSQL solutions facilitate handling large volumes of unstructured data, which is particularly relevant for dynamically evolving web applications.

In TefDB development, PostgreSQL was selected because of its reliability, performance, and support for complex biological data structures. The database schema was designed to store integrated tef datasets, which included literature, genome, gene, nucleotide, GEO, PubChem, and taxonomy data.

### Environment configuration and dependency management

Environment-specific configurations were handled through **.env** files to securely handle database credentials and API keys, which ensured that the application behaved the same way in development, testing, and production environments.

#### API design and data access

The system provides RESTful APIs for frontend-backend interaction or communication, including searching, filtering, creating, updating, and retrieving data, with standardized response formats, input validation, and exception handling.

#### Security and user management

The Python programming language ensures fault tolerance and security. In addition, Django incorporates security features such as authentication and authorization, role-based access control, secure handling of user submissions and uploaded datasets, and protections against data tampering and sensitive operations being performed by unauthorized users.

### Frontend design and development

The platform was designed for a user-friendly experience, with a focus on responsiveness and accessibility. The interface design emphasized intuitive navigation, consistent layouts, and minimal cognitive load, achieved through the use of HTML, CSS, and JavaScript stack technologies. The dynamic frontend was built with React.js, ensuring a reactive and component-based architecture.

#### Use case diagrams

**Figure 4:**
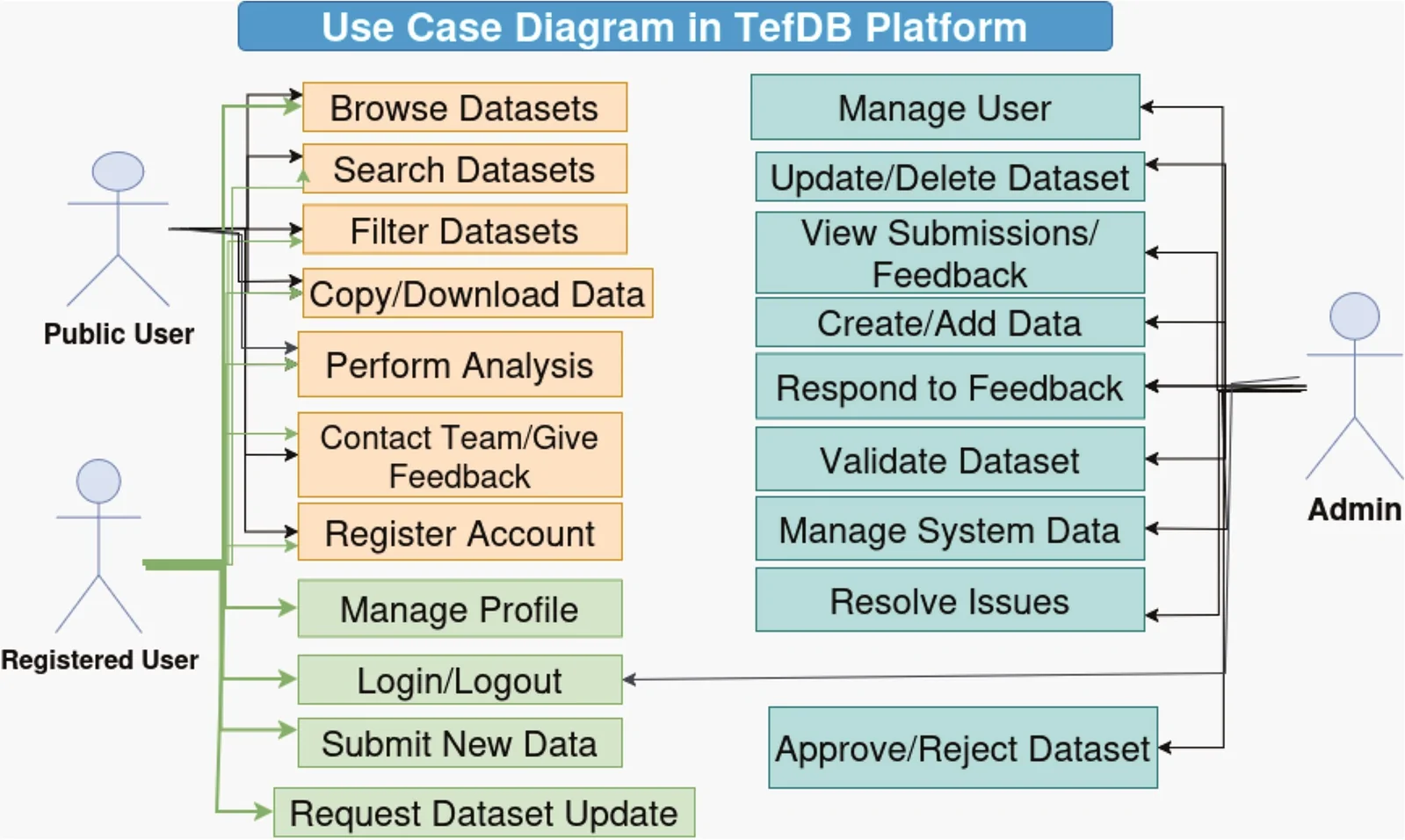
Use-case diagram of TefDB showing interactions between actors (Public User, Registered User, and Administrator) and system functionalities, including data browsing, analysis, submission, authentication, and feedback.

A use case diagram is a visual way to show how users (actors) interact with a system and what functions (use cases) the system provides (see Fig. 4).

#### Purpose of the use case model

The use case model shows the interactions between users (actors) and the platform (system) (Fig. 4), with a detailed summary of actors, preconditions, post-conditions, and success scenarios provided in (Table 7) . It gives an overview of system functions and helps stakeholders understand how the platform supports the management and analysis of *Eragrostis tef* resources.

**System Actors:** Public user, Registered user, and Admin

**Administrator:** Responsible for managing datasets (deleting, updating, creating, etc.); overseeing user accounts; monitoring the user’s feedback and contact; approving the new data submitted; and maintaining the system.

**Registered User** (Researcher, student, *etc*.): Authenticated users who can submit new datasets, explore, analyze, and download datasets.

**Public User**: Unauthenticated users who can browse or access data, send feedback, and do other activities but cannot submit or upload new data submissions.

**Table 7:** Summary of the use-case model of the platform, showing actors, preconditions, post-conditions, and success scenarios for each use case (UC01–UC06) (Table 7).

Use case	Actor	Precondition	Post-condition	Success scenario
**UC01:** Upload data	Admin	Dataset file available	Data stored in DB	Admin uploads & system validates
**UC02:** Authentication	Registered User	User registered	User logged in	User logs in successfully
**UC03:** Browse Data	Registered/Guest	Dataset exists	Results displayed	User searches & sees results
**UC04:** Analysis	Registered/Guest	Dataset exists and integrated or linked tools	Visualization generated	User runs analysis
**UC05:** Feedback	Any user	Form available	Stored in DB	Feedback submitted
**UC06:** Submit new data	Registered User	Form available	Stored in DB	Data submitted

It identifies the actors involved in each function, the required preconditions before an action is performed, the expected post-conditions after the process is completed, and the typical success scenarios. The table highlights the core functionalities of the platform, including user authentication, dataset uploading, browsing and analysis of data, submission of new datasets, and user feedback. Overall, it provides a clear overview of how the system supports data access, management, and user participation.

#### Activity diagrams

**Figure 5:**
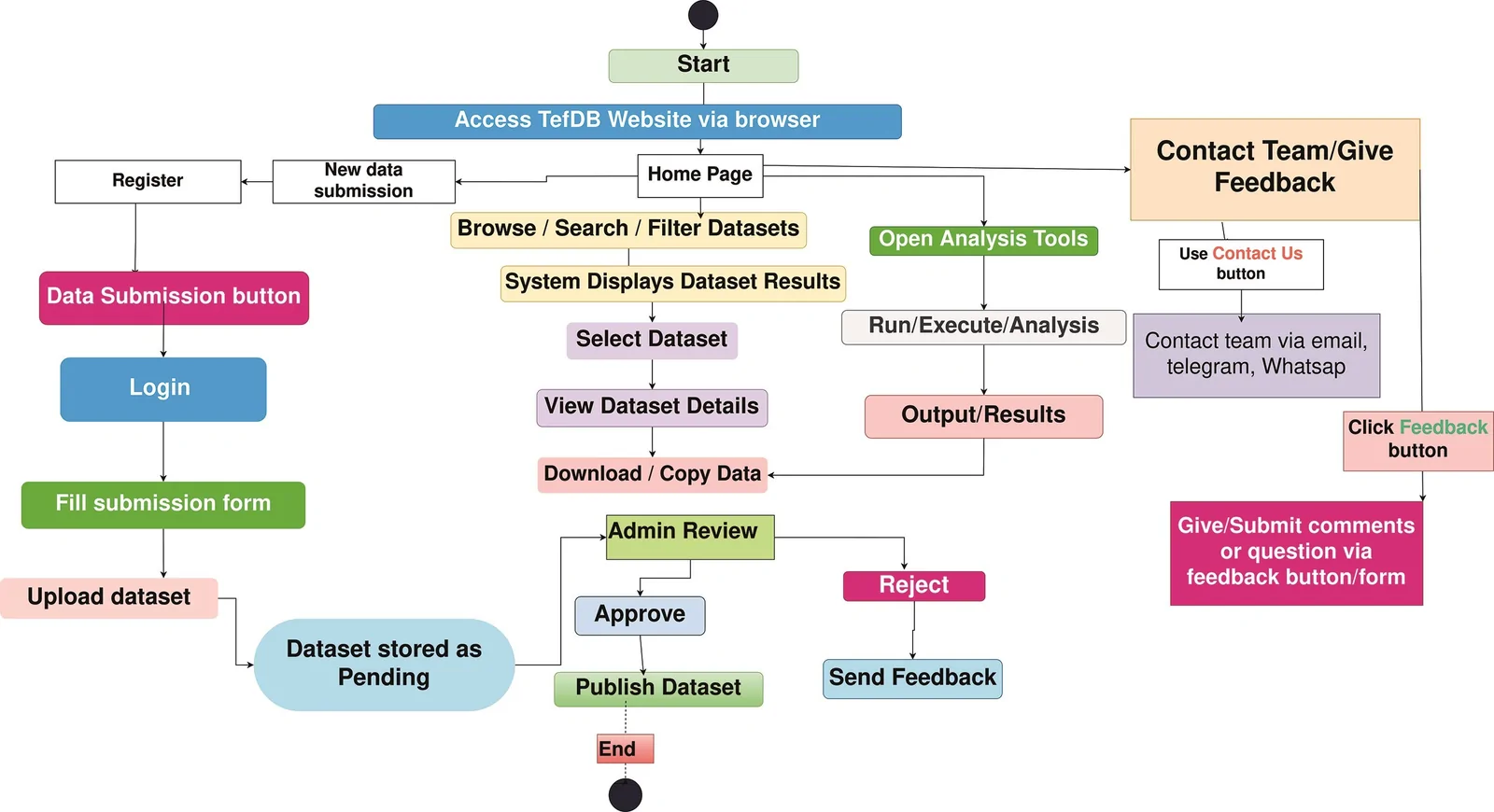
Activity diagram of TefDB illustrating the workflow for data browsing, searching, filtering, downloading, analysis, and dataset submission with an administrator approval process.

An activity diagram is essentially *an advanced version of a flow chart* that models the flow from one activity to another activity (see Fig. 5).

An activity diagram illustrates the workflow in the TefDB system. Users can browse, search, and filter datasets and perform operations such as downloading, copying, analysis, and visualization. Registered users can submit new datasets through the submission form. The submitted datasets are stored by the system and reviewed by the administrator. After verification, the administrator either approves and publishes the dataset in the database or rejects it and sends feedback to the user.

#### Frontend implementation

The TefDB frontend was implemented as a web-based, user-friendly, and interactive interface to facilitate the data accessibility, analysis, and usability of *Eragrostis tef* resources. Nevertheless, the frontend is component-based to allow for modular development and interface component reuse and supports dynamic data rendering, interactive visualizations, and communication with backend APIs. During development, cross-browser and device compatibility were taken into consideration to ensure maximum accessibility.

#### Component-based architecture

The frontend follows a component-based design by React.js frameworks, where each bioinformatics dataset and tool is represented as an independent React component.

**Major components include the** Gene Component, Nucleotide Component, Protein Component, Genome Assembly Component, Taxonomy Component, PubMed Component, BioProject Component, *etc*. This approach improves code reusability, simplifies debugging, and supports future expansion of the platform.

### Bioinformatic tools integration

A major advantage of TefDB is the integration of many common bioinformatics analysis tools into a single web interface.

#### Integrated tools into TefDB

The development environment was further configured to enable seamless integration with essential bioinformatic tools, supporting computational analyses and visualization. Tools were either installed locally on the server or accessed remotely through their web addresses, depending on the requirements and system compatibility. Necessary system dependencies, command-line tools, and server configurations were implemented to ensure efficient communication between the TefDB platform and these bioinformatics resources. Table 8 shows the integrated tools into the TefDB system.

**Table 8:** Bioinformatic tools integrated into TefDB, including BLAST (local installation), JBrowse 2 (local installation), Primer3Plus (external link), and Clustal Omega (external link), with their purposes.

Item	Tool/platform	Installation/access method	Purpose	Description
1	BLAST	Local Installation	Sequence alignment & sequence similarity search	Installed locally to enable fast, reliable nucleotide and protein sequence searches against integrated databases.
2	JBrowse 2	Local Installation	Genome visualization	Deployed locally to provide interactive genome browsing and visualization within the platform.
3	Primer3Plus	External web link	Primer design	Accessed via its web address to allow users to design primers for PCR experiments.
4	Clustal Omega	External web link	Multiple sequence alignment	Integrated through its online service to perform sequence alignments and phylogenetic analysis.

#### BLAST (basic local alignment search tool)

**Figure 6:**
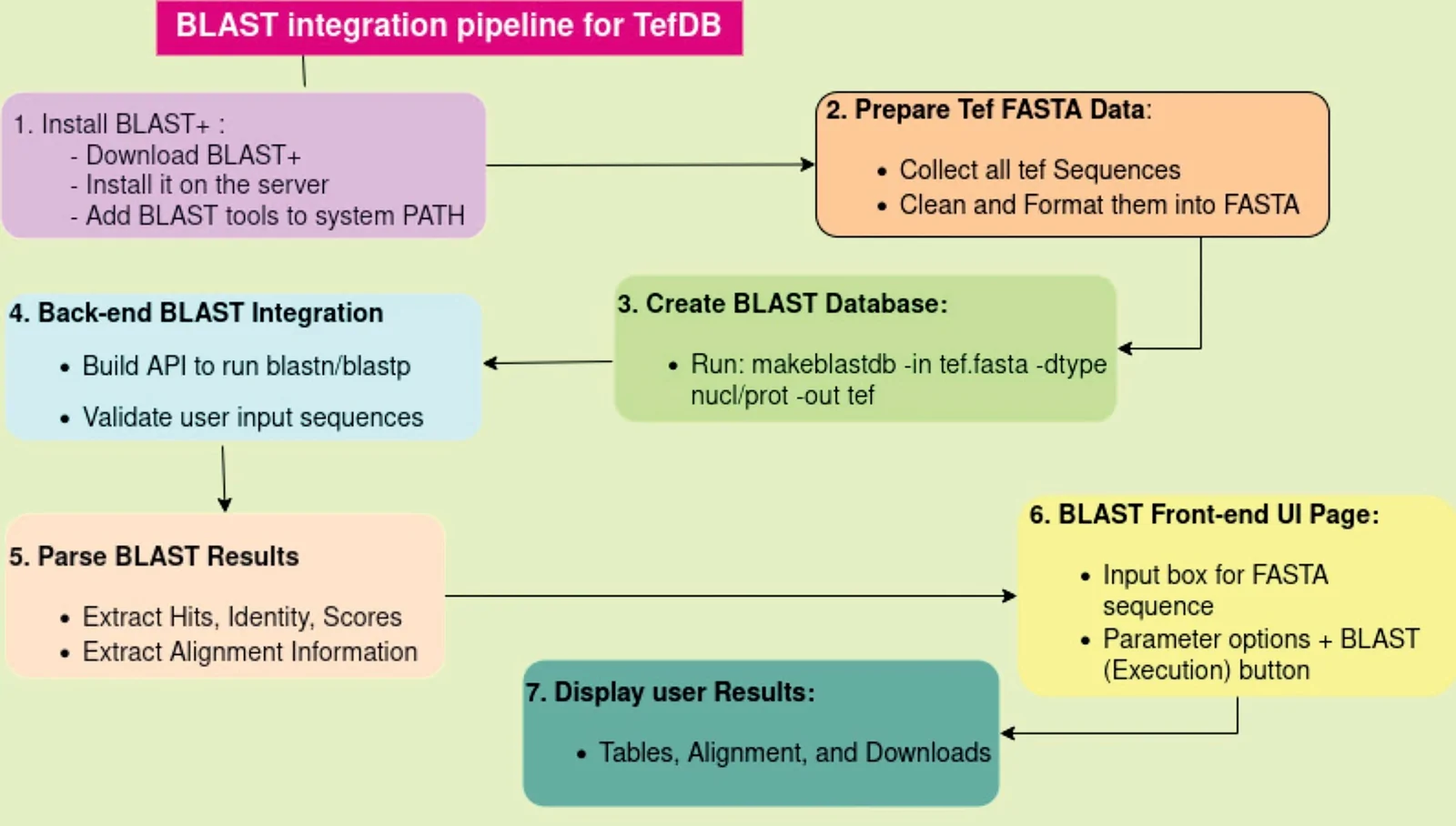
BLAST integration pipeline showing the workflow from user query submission to local database search and result visualization within the TefDB platform.

The BLAST was integrated to TefDB platform to enable the fast, automated sequence comparison against local databases, with NCBI-BLAST linked for further analysis. It was integrated through the following pipelines. Fig. 6 shows the pipeline of the BLAST TefDB integration.

#### JBrowse 2

**Figure 7:**
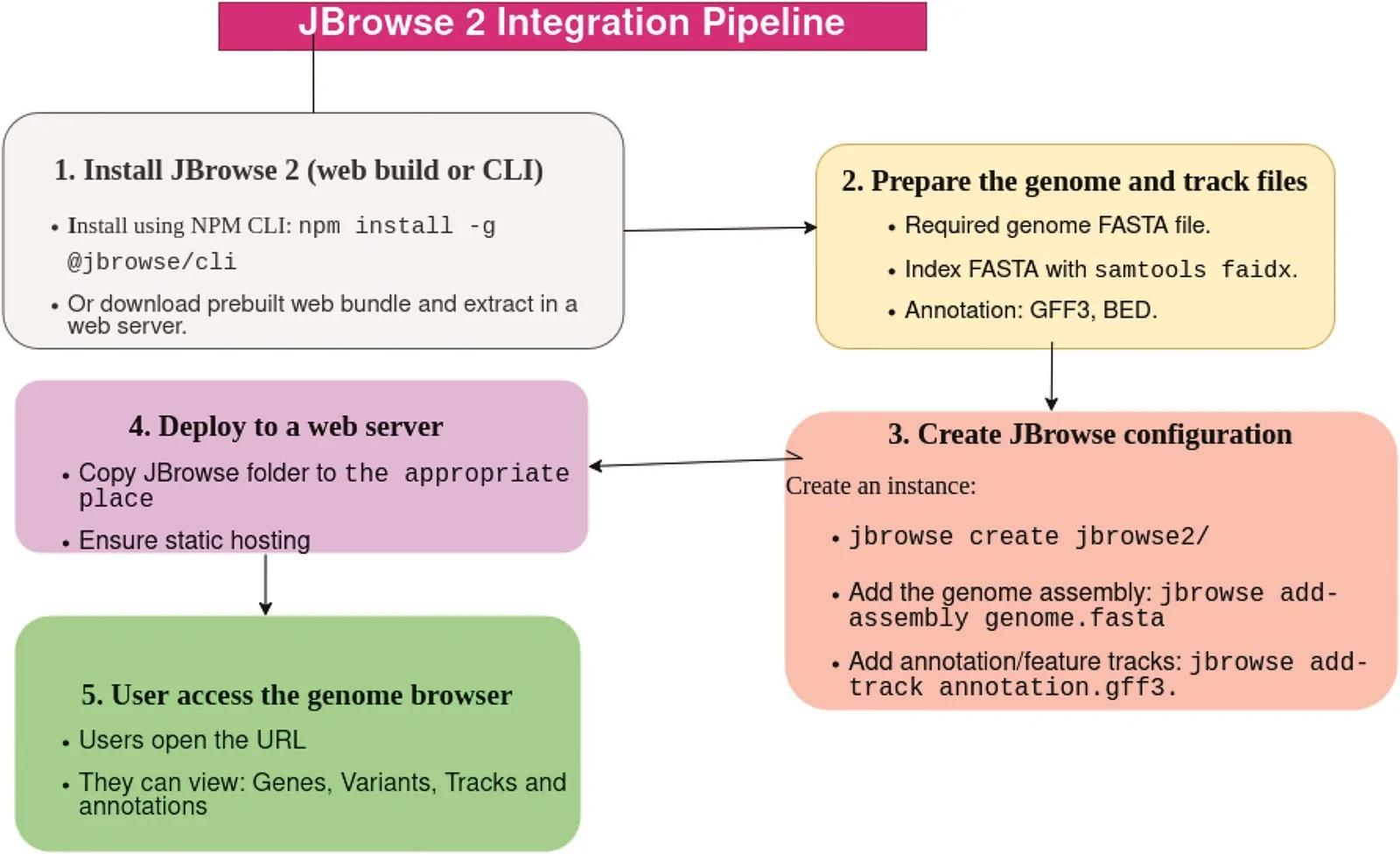
JBrowse 2 integration pipeline depicting the process of preparing genome data, configuring the genome browser, and rendering interactive genomic visualizations.

JBrowse 2 offers an interactive genome browser that visually represents gene annotations, variants, and genomic features through a user-friendly web interface. It facilitates the rendering of large-scale genomic data in scalable views of chromosomes, exons, introns, and regulatory elements. In TefDB, JBrowse 2 is employed for the visualization of the structure and organization of *Eragrostis tef* gene annotations, variants, and genome assemblies. This is intended to help researchers in exploring genomic regions of interest, inferring context on gene location, and performing further comparative genome studies in a more user-friendly manner. The step or pipeline in which the JBrowse 2 integrated was shown in Fig. 7.

#### Primer3Plus

Primer3Plus is an online primer design tool linked to TefDB through the link address (https://www.primer3plus.com/index.html) into the platform that designs PCR primers according to the sequences provided by the user. The purpose is to enable the experimental validation of genes, the study of gene expression, or the development of genetic markers.

#### Clustal Omega

Clustal Omega is linked to TefDB through the link address (https://www.ebi.ac.uk/jdispatcher/msa/clustalo) for the purpose of multiple sequence alignment, allowing the comparison of nucleotide or protein sequences. It aligns homologous sequences in order to find conserved motifs, evolutionary relationships, and functional domains. Through TefDB, Clustal Omega allows alignment of sequences across different assemblies or against orthologs derived from related species as a means of phylogenetic analysis, functional annotation, and identification of conserved regions suitable for primer design or molecular studies.

#### Integration approach

The web interface provides access to each tool, enabling users to submit inputs, run analyses, and view results without ever leaving the platform. Integration was intended to provide the following:

- Consistent data formats
- Efficient execution
- Clear visualization of outputs

This approach increases analytical capability while minimizing workflow fragmentation.

### Iterative development and refinement

The system was developed iteratively, with refinements based on testing outcomes and user feedback, and addressed issues in navigation, performance, and data filtering across successive development cycles to create a more robust and usable platform.

In this chapter, we described how the TefDB platform was designed and developed, from the system architecture to the database modeling, backend and frontend implementation, and integration of bioinformatics tools.

## Results

This section presents the results obtained from the development of the knowledge-based *Eragrostis tef* platform, called TefDB. The results include survey findings for requirement analysis, dataset integration outcomes, API development, and analytical tool availability. These results show how the system meets the functional, performance, usability, and analytical needs of researchers working with *Eragrostis tef*.

### Survey results for requirement analysis

**Figure 8:**
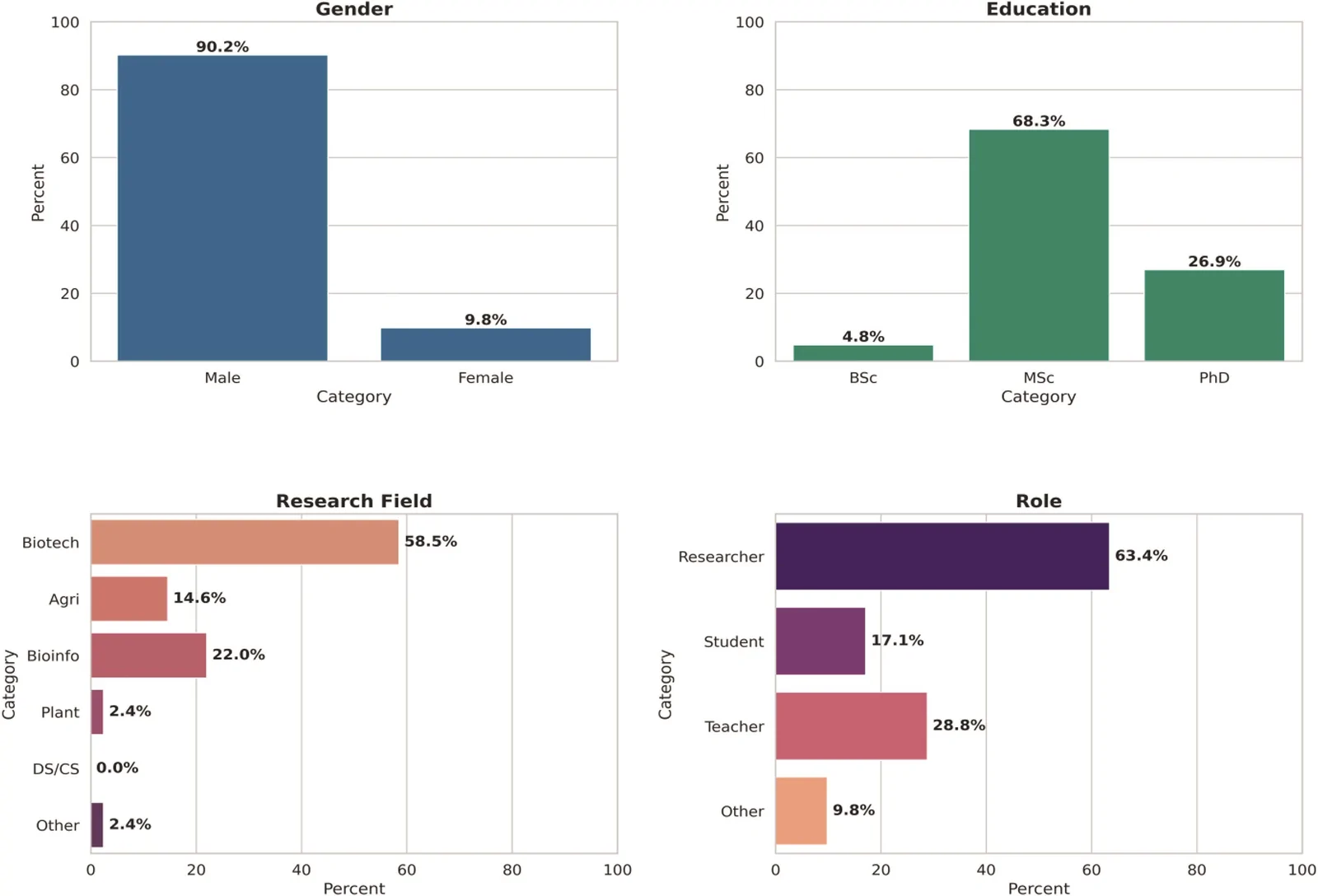
Respondent profile of the user survey (n = 41), showing distribution by gender, educational level, research field, and professional role.

A survey was conducted among 41 participants, including researchers, students, educators, and bioinformatics practitioners, to identify both functional and non-functional requirements for the proposed TefDB platform. The survey collected information on demographics, experience with *Eragrostis tef*, data usage, preferred database features, and expectations for a species-specific bioinformatics platform (Figure 8). The findings were used to guide system design, dataset integration, and tool selection.

### Respondent profile

A gender imbalance was noted in the sample. In terms of educational attainment, most respondents held a Master's degree (68.3%), followed by those with a PhD (26.9%), and a small proportion possessed a Bachelor’s degree (4.8%). The sample included both senior professionals (63.4% researchers) and academic trainees (17.1% students).

Regarding primary research fields, biotechnology constituted the largest proportion (58.5%), followed by bioinformatics (22%) and agriculture (14.6%). Other fields, including plant biology and miscellaneous areas, were minimally represented (each 2.4%), and no respondents reported a background in data science or computer science. This distribution indicates a strong concentration in the life sciences, particularly biotechnology, with limited interdisciplinary representation.

Concerning professional roles, the sample comprised both senior professionals (63.4% researchers) and academic trainees (17.1% students). Overall, the findings suggest that the respondent group consists largely of highly educated professionals engaged in research within biotechnology and related life science disciplines. However, the limited diversity in gender and disciplinary background should be considered when interpreting and generalizing the results of this study.

### Data preferences and needs

Respondents were asked about the types of biological data they currently use and would like to access through TefDB. Participants could select multiple options; percentages therefore sum to > 100% and represent the proportion of respondents who selected each option.

**Figure 9:**
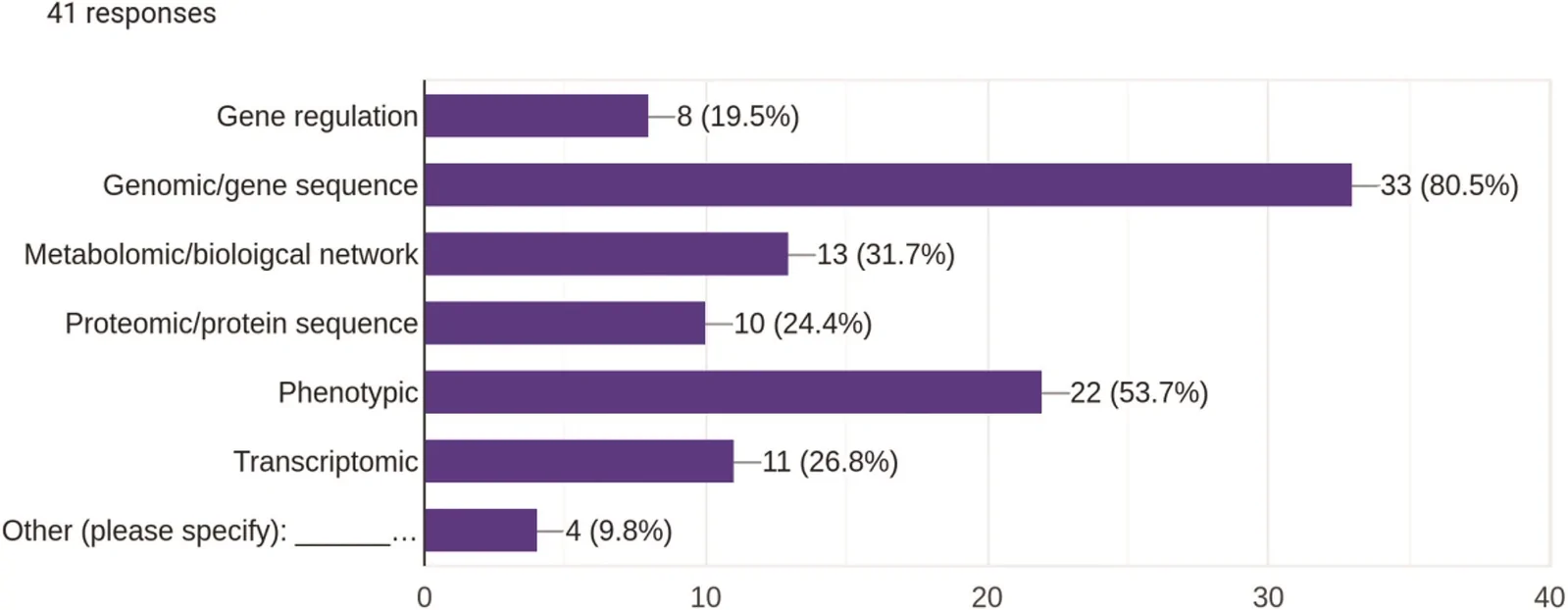
Respondent preferences for data types, indicating the percentage of participants who work with genomic, phenotypic, proteomic, and other biological data types.

As shown in Fig. 9, most respondents reported working with genomic or gene sequence data (80.5%), followed by phenotypic data (53.7%). This indicates a strong demand for integrated genomic and phenotype-related datasets within the platform.

### Bioinformatics tools familiarity and requests

**Figure 10:**
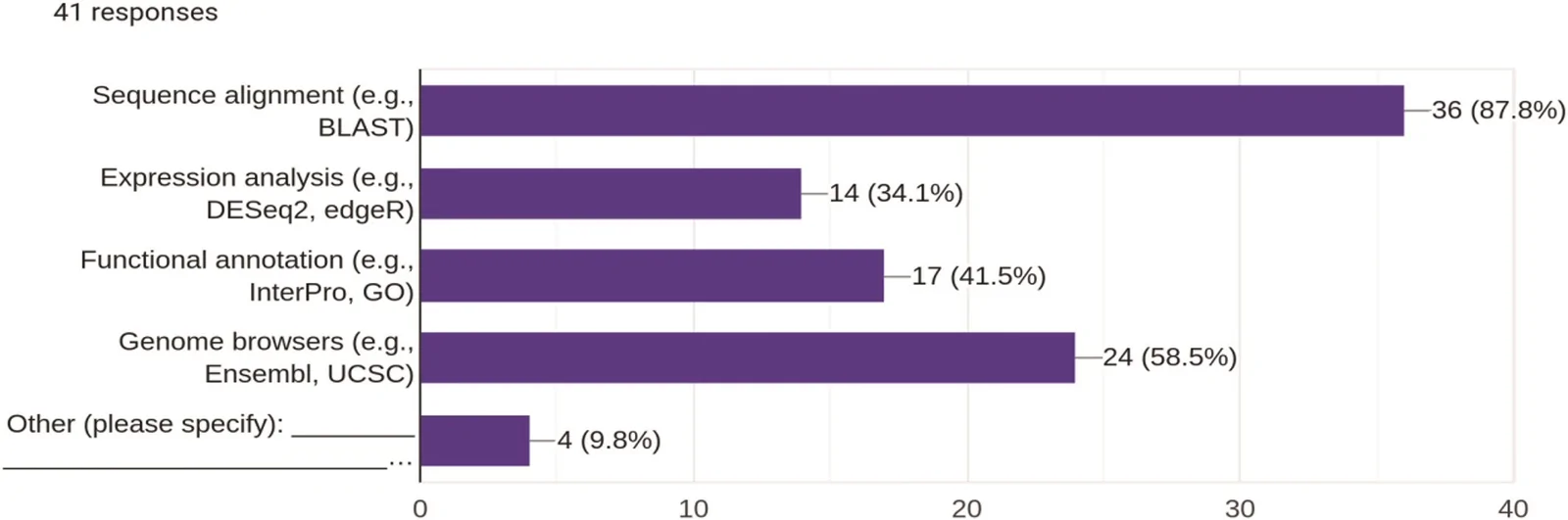
Familiarity with and requested bioinformatics tools, showing BLAST and genome browsers as the most commonly used tools among respondents.

The survey also evaluated respondents’ familiarity with bioinformatics tools and their preferences for integration into TefDB. As illustrated in Figure 10, the most commonly used and requested tools were BLAST (87.8%) and genome browsers (58.5%). Participants could select multiple options; percentages therefore sum to > 100%.

**Figure 11:**
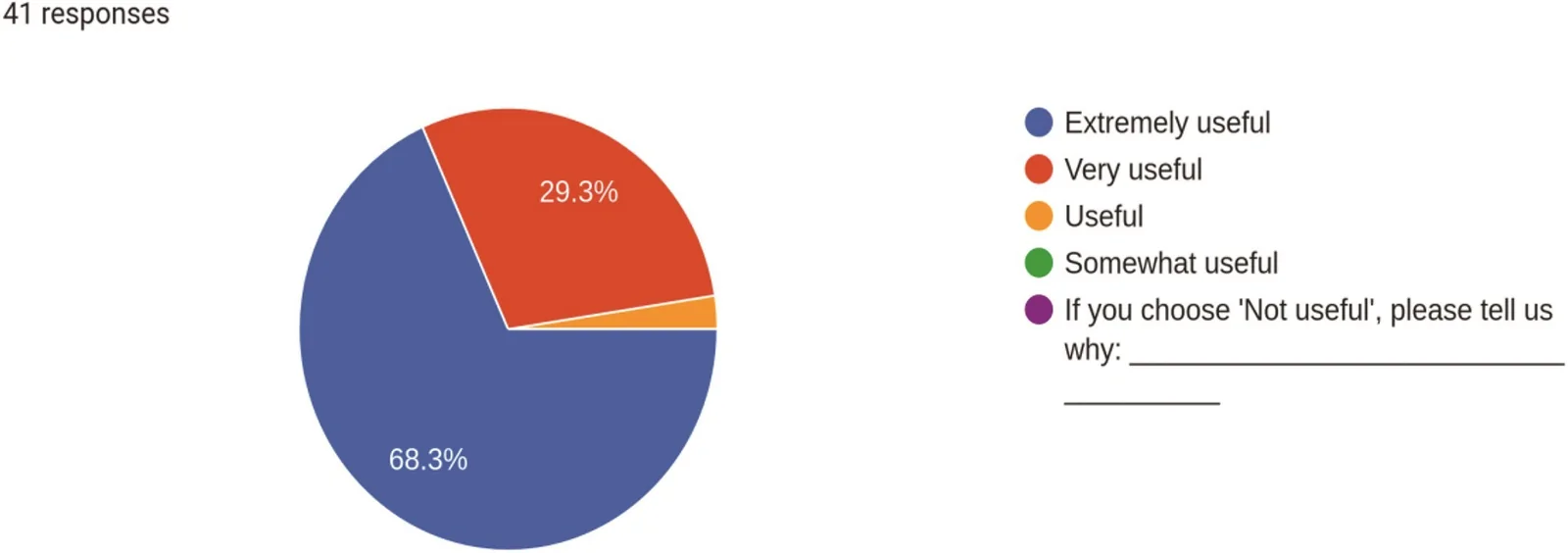
Perceived usefulness of a dedicated *Eragrostis tef* database with integrated bioinformatics tools (100% of respondents found it useful).

Furthermore, as shown in Figure 11, all respondents (100%) expressed interest in the development of a dedicated database with integrated bioinformatics tools specifically for *E. tef*, as it is useful. However, it should be noted that this question was positively framed, which may have introduced an optimism bias. This finding highlights a clear gap in the availability of existing resources.

**Figure 12:**
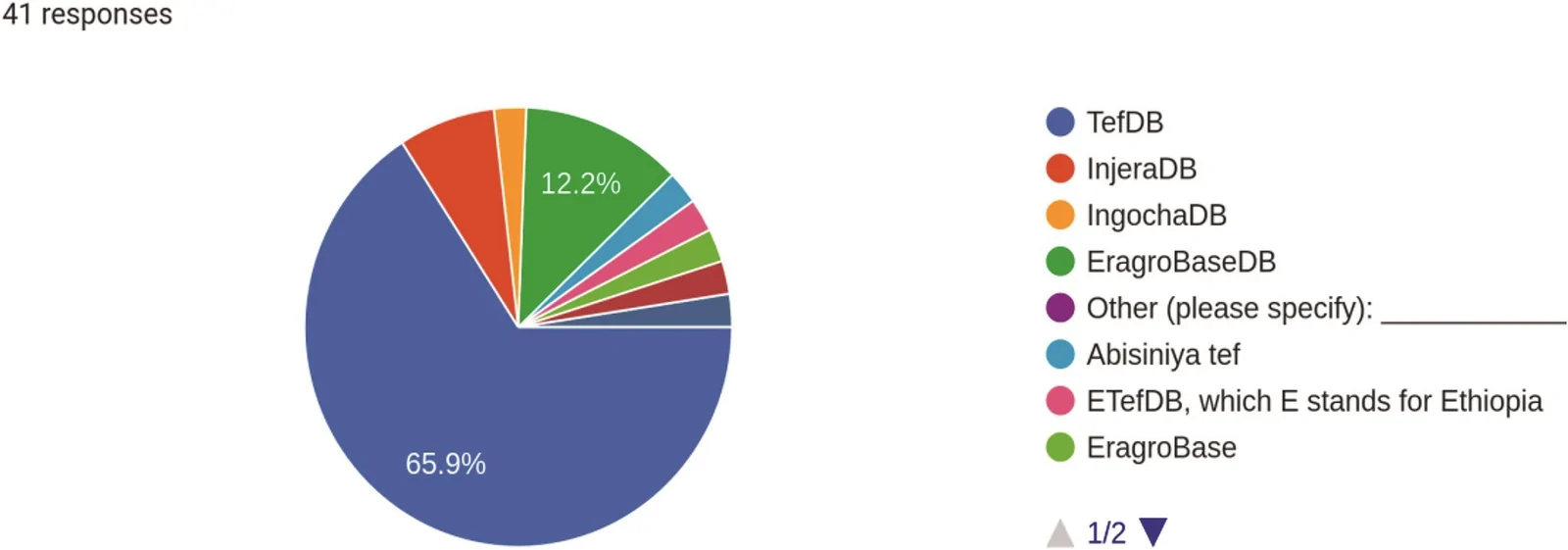
Preferences for naming the Eragrostis tef database, with TefDB receiving the highest support (65.9%).

Respondents were also asked to suggest or select a name for the proposed database. As presented in Figure 12, 65.9% of respondents preferred the name *TefDB*. Based on this majority preference, the system has been named *TefDB*.

### Challenges with existing platforms

Respondents reported several limitations associated with existing platforms used for *Eragrostis tef* research. The most commonly identified challenges included limited data accessibility and fragmentation of data across multiple repositories. Participants also indicated the absence of a species-specific database for *E. tef*. In addition, limited awareness and communication among researchers, as well as insufficient collaboration tools, were reported.

### User expectations for TefDB

Respondents identified several key expectations for the proposed TefDB platform. These included the need for a user-friendly and open-access system, integration of bioinformatics tools with comprehensive datasets, and efficient data management capabilities. Participants also emphasized the importance of providing high-quality and up-to-date data, as well as continuous expansion of available data. Overall, respondents indicated that the platform should support and facilitate research activities related to *Eragrostis tef*.

### System implementation results

**Figure 13:**
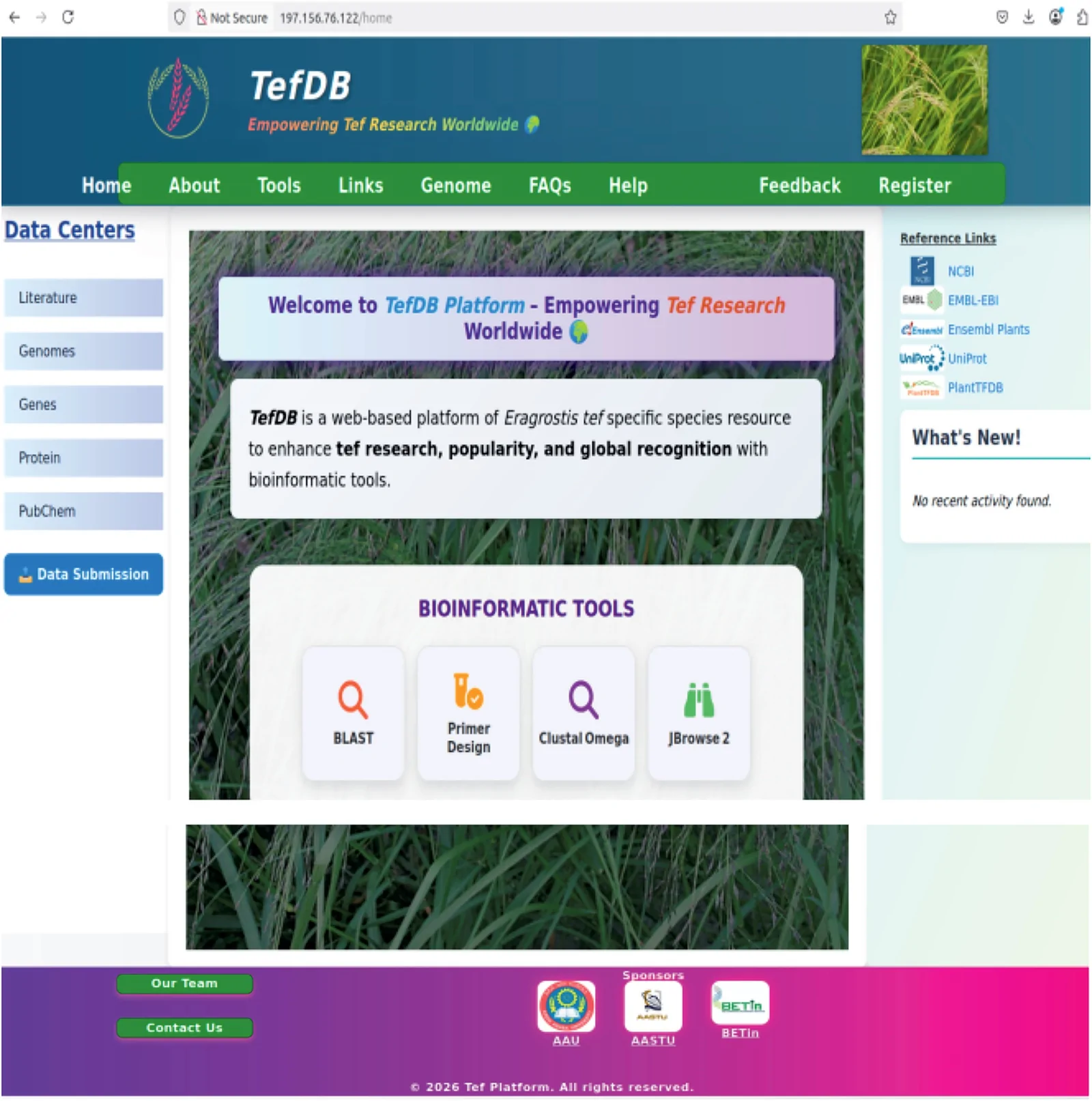
User interface of the TefDB platform, showing the homepage with navigation menu, search functionality, and data categories. JBrowse user interface page.

The primary outcome of this study is a functional web-based bioinformatics platform focused on *Eragrostis tef*, referred to as TefDB. The system was developed through the integration of frontend technologies (HTML, CSS, JavaScript, and React.js) with backend frameworks (Node.js and Django), supported by a PostgreSQL database on an Ubuntu 22.04 LTS operating system. The user interface of the platform is presented in Fig. 13.

**Figure 14:**
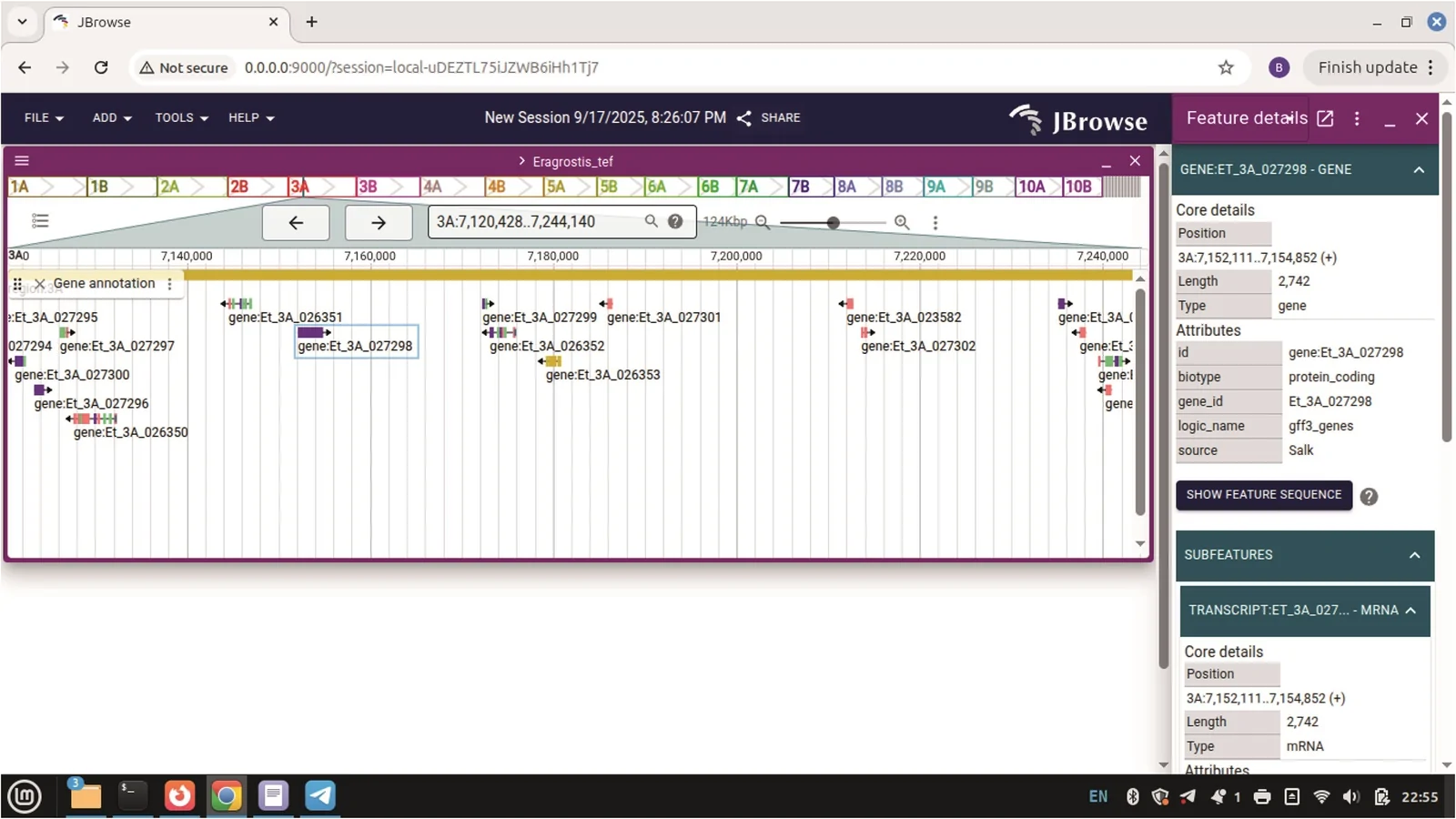
JBrowse user interface page within TefDB, displaying the genome visualization component with gene annotations and sequence variants.

The JBrowse user interface page presents the genome visualization component of TefDB. TefDB is a biological database for *Eragrostis tef* integrated with JBrowse 2, a web-based genome browser that enables users to visualize the reference genome along with associated gene annotations and sequence variants (see Fig. 14). This integration facilitates interactive exploration and interpretation of genomic data within the platform.

### BLAST user interface page

**Figure 15:**
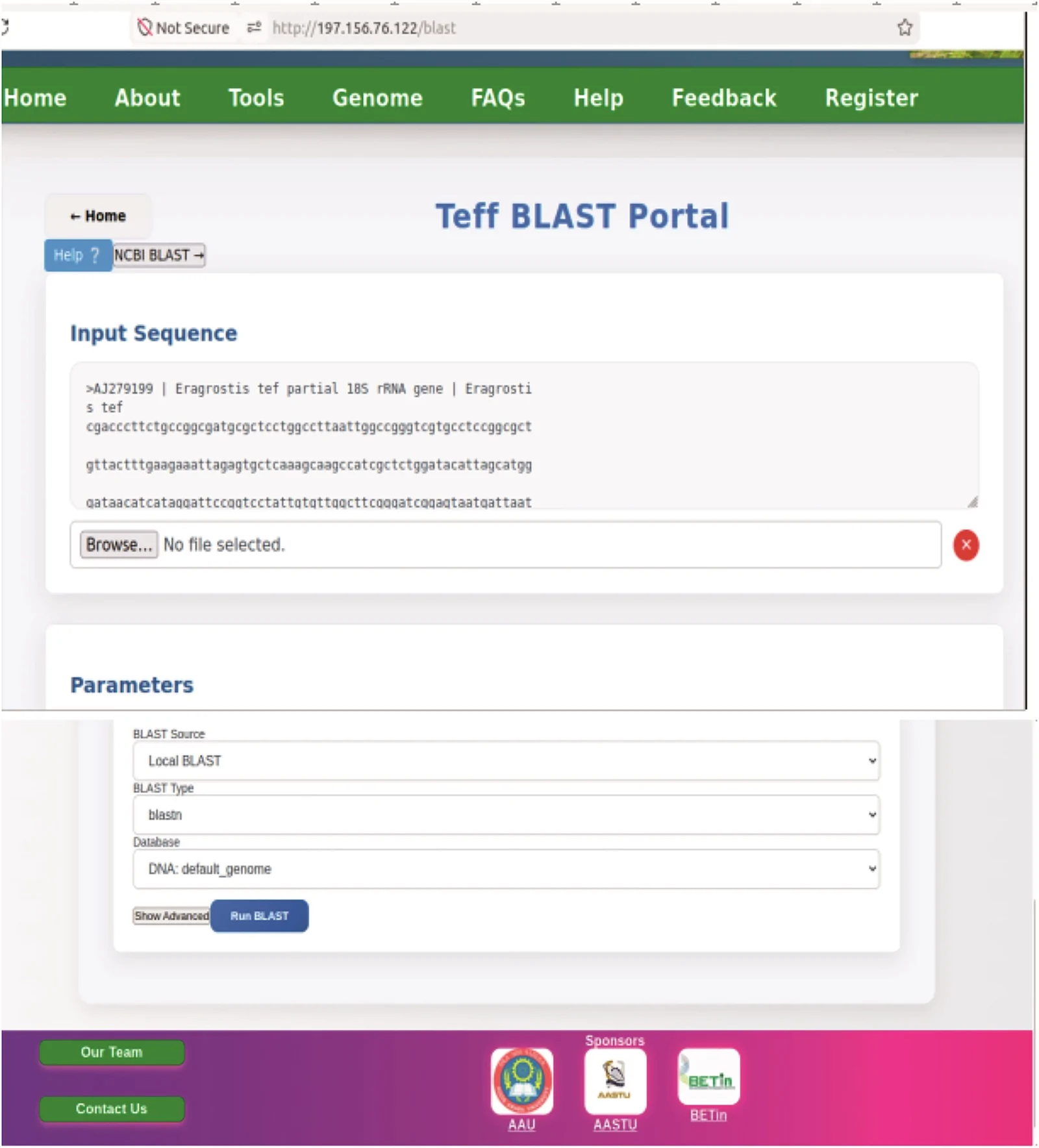
BLAST user interface page showing the sequence similarity search interface with query input options and result display.

The BLAST user interface page provides sequence similarity search functionality within TefDB. BLAST is a widely used tool for comparing nucleotide or protein sequences against sequence databases and evaluating the statistical significance of matches. Within the platform, users can query nucleotide or protein sequences against local *E. tef* genome datasets or access external databases such as NCBI BLAST for broader analysis (see Fig. 15). This functionality supports the identification of homologous sequences, detection of gene orthologs, and inference of functional relationships within biological data.

### Functional API testing results

All API endpoints returned valid JSON responses with status code 200. The response format complied with the specified API structure, and every test executed successfully without errors or inconsistencies. These results confirm the stability and functional integrity of the TefDB platform. The test configuration and results are summarized in Table 9.

**Table 9:** Results of functional testing using Postman, showing 320 requests executed with 0% errors, average response time of 127 ms, and total execution duration of 29 seconds.

Execution date	Jun 15, 2026 01:45:47
Test environment	none
Number of iterations	10
Total requests executed	320
Total execution duration	29s 125ms
Average response time	127 ms
**Failed, skipped, or erroneous tests**	**0**

The following conditions were verified for each request using automated test assertions:

- The status code for the HTTP response was 200 (**OK**).
- The body of the response was sent back in a valid JSON format.
- The response format complied with the specified API structure.
- Every test that was run was successful and free of errors or inconsistencies.

These outcomes shows that the TefDB platform reliably generates accurate answers during multiple executions. As a result, the implemented API services' stability and functional integrity are verified.

### Performance testing results

The performance test ran for 10 minutes with 20 virtual users, sending 4 870 requests. The system achieved a throughput of 32.48 requests per second, with an average response time of 491 ms and an error rate of 0.00%. These results demonstrate that the platform can efficiently handle moderate concurrent access. The performance test was conducted using a fixed load profile, with the key parameters and results summarized in Table 10.

**Table 10:** Results of performance testing using Postman, showing 4 870 requests with 20 virtual users, throughput of 32.48 requests/second, average response time of 491 ms, and 0% error rate.

Test start time	Jun 15, 2026 10:10:45 (10 mins)
Total test duration	10 minutes
Load profile	Fixed
Test environment	none
Number of virtual users	20
Total requests sent	4 870
Throughput	32.48 Requests/second
Average response time	491 ms
**Error rate**	**0.00%**
**Failure**	**0.00%**

Table 10 Throughout the entire test duration, the TefDB platform maintained stable throughput and consistent response times, with no recorded errors or failed requests. The observed average response time remained within acceptable limits for a data-intensive bioinformatics system, indicating that the platform can efficiently handle moderate concurrent access.

### Usability results

Questionnaire responses indicated that users were satisfied with navigation, interactivity, performance, and responsiveness. The Help & User Guide page reduced task completion time for new users. All major interface components (navigation menu, data centers, authentication, and feedback pages) functioned correctly without broken links or inconsistencies.

**Figure 16:**
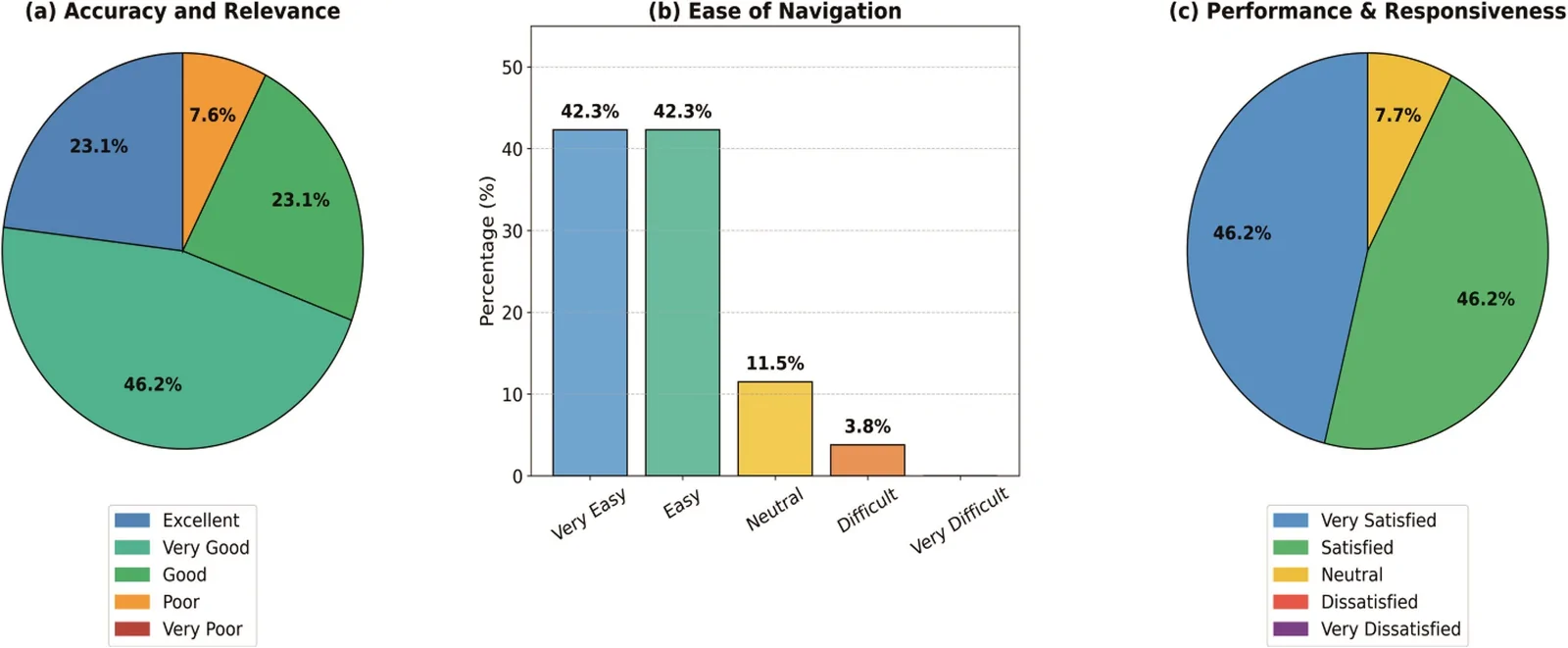
Usability evaluation results showing: (a) data accuracy and relevance ratings, (b) ease of navigation ratings, and (c) system performance and responsiveness satisfaction level.

As shown in Fig. 16 (a–c), the results indicate that the data accuracy and relevance, ease of navigation, user interface friendliness, and system responsiveness were positively evaluated by the majority of users.

As shown in Fig. 16a, the majority of respondents rated the accuracy and relevance of the data positively, with 46.2% indicating the data quality as *very good*, while 23.1% rated it as *excellent* and another 23.1% as *good*. Only a small proportion of users (7.6%) reported *poor* data quality, and no respondents selected *very poor*, indicating overall strong confidence in the data provided by the platform.

As illustrated in Fig. 16b, system navigation was also evaluated favorably. Most participants found the platform easy to use, with 42.3% rating it as *very easy* and 42.3% as *easy*. A small percentage (11.5%) expressed a neutral opinion, while only 3.8% found it difficult to navigate. These results suggest that the interface design effectively supports efficient access to datasets and platform functionalities.

Similarly, Fig. 16c shows that users expressed high levels of satisfaction with system performance and responsiveness. Specifically, 46.2% of respondents reported being *very satisfied*, and an equal proportion (46.2%) indicated they were *satisfied*, with only 7.7% remaining neutral and no negative responses recorded.

Overall, these findings demonstrate that the TefDB platform provides a reliable, efficient, and user-friendly environment for accessing and exploring biological data related to *Eragrostis tef*.

### Dataset integration results

The TefDB platform successfully integrated multiple categories of *Eragrostis tef* data from various sources, including genomic sequences, gene and protein information, literature, and chemical/biological datasets. Table 11 summarizes the dataset coverage in the platform.

**Table 11:** Summary of integrated Eragrostis tef datasets, showing 77 795 total records across literature (1 372), genome (6 144), gene (66 455), protein (3 447), and chemical/pathway (377) data categories.

Data category	Dataset type	Total records in each data center
Literature	PubMed (203), PMC (1167), Bookshelf (2)	1 372
Genomes	Assembly (2), BioProject (23), BioSample (1273), Nucleotide (3683), SRA (1162), Taxonomy (1)	6144
Genes	Gene (66 419), GEO (36)	66 455
Proteins	Protein (3 447)	3 447
PubChem	Pathway (372), BioAssay (1), Substance (4)	377
**Total**		**∼ 77 795** datasets

### API implementation results

All datasets in TefDB are accessible via RESTful API endpoints, which return structured JSON responses to support programmatic access. API testing was conducted using both manual requests and automated tools (Postman and Selenium) to verify correct routing, response structure, and consistent outputs across different query types. The tests confirmed that the endpoints function as expected, enabling integration with external tools and workflows. This implementation allows external tools, scripts, and automated workflows to retrieve and interact with the platform’s data efficiently, enhancing flexibility and enabling integration with other bioinformatics applications.

### Documentation and user support results

TefDB provides user support through a help & user guide and API documentation (http://197.156.76.122/help), FAQs (http://197.156.76.122/faqs), and feedback modules (http://197.156.76.122/contact). These resources improve platform accessibility and facilitate user navigation. The usability survey and system testing indicate that these modules effectively guide users, contributing to the platform’s overall goal of providing a centralized, interactive, and analytical resource for *Eragrostis tef* research.

## Discussion

The *Eragrostis tef* knowledge-based platform, TefDB, facilitates accessibility, management, and analysis of tef resources. This section discusses selection of technology, the developed platform is compared with existing databases, the system strengths, limitations, and the broader implications of the platform.

### Interpretation of Key Findings

The results of this study demonstrate the successful development of TefDB, a centralized, integrated bioinformatics platform dedicated to *Eragrostis tef*. The platform integrates diverse biological data types (genome, gene, protein, nucleotide, literature), bioinformatics analysis tools (BLAST, JBrowse 2, Primer3Plus, Clustal Omega), and links to external references (NCBI, EnsemblPlants, UniProt, EMBL-EBI) within a single environment. This integration significantly enhances tef-related data accessibility and analysis while reducing the need for researchers to navigate multiple external databases.

The integration of searching, browsing, filtering, and data retrieval functions enables efficient exploration of *E. tef* resources. The platform simplifies research workflows by keeping data and analysis tools co-located. Public users can freely view, search, and analyze data and provide feedback without registration, promoting open access. Registered users can submit new datasets, contributing to continuous expansion of tef bioinformatics resources.

Testing and evaluation demonstrate high user satisfaction (see Figs. 16a-c), with functional testing (Table 9) showing 0.00% errors and performance testing (Table 10) confirming stable response times under concurrent usage (20 virtual users). These findings demonstrate that TefDB has the potential to serve as an integrated platform for managing and analyzing biological data for *Eragrostis tef* research. This work also illustrates the advantages of organism-specific, centralized database systems for biological data management.

### Importance of a tef-specific database

TefDB provides a specialized platform dedicated to *Eragrostis tef*, addressing a significant gap in available bioinformatics resources. While general databases such as NCBI, UniProt, and Ensembl Plants include tef data, they are not tailored to the unique resources research needs of this crop. TefDB, by integrating biological datasets including genomic, nucleotide, protein, literature, *etc.*, and linked bioinformatic tools within a single platform, enables researchers to efficiently retrieve, visualize, and analyze tef-specific data. This focused approach facilitates local and regional research, supports the design of experiments, and improves the interpretation of genomic and phenotypic information. Moreover, the platform streamlines workflows by reducing the need to switch between multiple external tools, saving time and enhancing productivity for tef research. The availability of a tef-specific database is therefore crucial for advancing genomic studies, crop improvement programs, and data-driven decision-making in tef research.

#### Strengths and innovations of TefDB

**Integration of diverse data and tools**: A primary strength of TefDB is the integration of various biological data types and analysis tools within a single platform, enabling streamlined, efficient workflows. The platform stores multiple tef-related resource categories (genome, gene, protein, nucleotide, literature, BioProject, BioSample, SRA), minimizing data fragmentation and improving retrieval and management.

**Bioinformatics tool integration**: The incorporation of BLAST (sequence analysis), JBrowse 2 (genome visualization), Clustal Omega (multiple sequence alignment), and Primer3Plus (primer design) enhances the platform’s practical value. TefDB serves as both a data repository and an active research support tool. Links to external references (NCBI, EnsemblPlants, EMBL-EBI, UniProt, & plantTFDB) improve interoperability with other biological repositories.

**Dual user access model**: TefDB supports interaction for both public and registered users. Public users can view and analyze datasets without registration, promoting open access to tef biological information. Registered users can contribute new datasets, enabling community-driven data expansion. The feedback/contact function facilitates communication between users and administrators.

**Modern technology stack**: Use of React.js, Django REST framework, PostgreSQL, Nginx, and Gunicorn ensures a scalable, maintainable, responsive system architecture.

### Data integration and system design considerations

A key strength of TefDB is the integration of multiple types of tef-related data within a single platform. This includes genomic sequences, gene and protein information, bio-project, bio-sample, literature, and pathway data from different public repositories such as NCBI, UniProt, and EnsemblPlants. The integration of these datasets provides researchers with a unified resource, reducing the need to navigate multiple external databases. Such a design enhances data accessibility, improves research efficiency, and supports comprehensive analyses of *Eragrostis tef*.

From a system design perspective, TefDB was developed using a modular architecture, which allows for easy expansion and addition of new datasets and tools. Relational database structures were employed to maintain data consistency and manage complex relationships among genomic, agronomic, and functional data. The user interface was designed to be intuitive, with clear navigation, search capabilities, and data visualization options, accommodating researchers with varying levels of technical expertise. Overall, the combination of data integration and thoughtful system design ensures that TefDB provides a reliable, user-friendly platform tailored to the specific needs of tef research.

### Bioinformatics relevance and contribution

The development of TefDB has multiple implications for tef research and bioinformatics applications. Tef is an important, nutritious, and economically valuable grain cultivated by millions of smallholder farmers in Ethiopia. The availability of comprehensive, centralized biological data within TefDB facilitates breeding programs, genetic studies, comparative genomics, and molecular biology research related to Eragrostis tef.

Data centralization. Unlike general repositories such as NCBI, UniProt, and EnsemblPlants, where tef-related data are scattered across multiple databases, TefDB consolidates 77 795 tef-specific biological records into a single, searchable platform, including 1 372 literature entries, 6 144 genome-related records, 66 455 gene models, 3 447 protein sequences, and 377 chemical and pathway entries. This centralization improves data exploration efficiency and reduces fragmentation that has historically hindered tef research.

Integrated analytical capabilities. TefDB integrates four bioinformatics tools to support research workflows. BLAST and JBrowse 2 are natively embedded, enabling sequence similarity searches and interactive genome visualization. Primer3Plus and Clustal Omega are externally linked, supporting PCR primer design and multiple sequence alignment for phylogenetic analysis.

Biological use cases. TefDB enables specific research workflows that are inefficient using general repositories. Researchers can access all tef bio-data in one place, discover available genomic data across 6 144 records, explore 66 455 gene models for drought tolerance candidates, retrieve 3 447 protein sequences for comparative studies, and examine 377 chemical and pathway entries. Additionally, users can perform sequence similarity searches against the nucleotide database and visualize results in JBrowse 2 to examine genomic context.

Broader implications. This work illustrates the significance of organism-specific databases for supporting specialized genomic research. TefDB demonstrates that a functional, centralized bioinformatics platform can be developed for an underrepresented crop species even with limited research funding. The expertise gained is transferable to platforms for other orphan crops such as finger millet, enset, and lablab.

#### System performance and reliability

The platform demonstrates effective performance for viewing, searching, and retrieving tef-related biological data. The data management system uses PostgreSQL as the backend database, with Django RESTful APIs connecting backend and frontend.

The React-based frontend provides good performance and user experience through dynamic data rendering and interactive navigation. Search and filter functions enable efficient retrieval of relevant biological data and literature. Pagination and category navigation improve usability and reduce complexity when working with large datasets.

Deployment using Nginx (web server) and Gunicorn (application server) contributes to stable server operation and efficient query processing. The RESTful API increases modularity and maintainability by separating frontend and backend. External linking mechanisms allow access to external repositories and analytical resources without unnecessarily duplicating datasets.

Evaluation results confirm effective platform operation. Functional testing showed all components function as expected with 0.00% errors (see Table 9). Consistent responses across different API endpoints indicate a well-structured, stable system. Performance testing under 20 concurrent users demonstrated stable throughput (32.48 requests/second) and consistent response times (491 ms average) with no errors (see Table 10), indicating TefDB is capable of managing typical research tasks.

### Limitations of the study

While the TefDB platform successfully integrates bioinformatic tools and supports dataset management and analysis for tef research, the study has several limitations. Some certain constraints are limited advanced bioinformatics tools, only the datasets available in public repositories were integrated, external tools (Primer3Plus, Clustal Omega) redirect users away from the platform, limited with international crop breeding databases, and performance may require optimization for very large genomics datasets. Additionally, authentication is implemented only for dataset submission, and advanced security features have not yet been incorporated. Future improvements could include cloud-based scalability, advanced visualization dashboards and analysis, and AI-driven data analysis modules.

The current TefDB deployment uses unencrypted HTTP (Fig. 13). While JWT authentication and CSRF protection are implemented in the Django backend code, the absence of HTTPS means that authentication tokens are transmitted in plain-text, making them vulnerable to interception. This limits the platform’s suitability for handling sensitive user data or confidential research information in its current state.

To address this limitation, we plan to implement SSL/TLS certificates and enable HTTPS in the next release, which will ensure secure transmission of authentication tokens and protect user data.

### Comparison with existing platforms

Many crop-specific databases have been created to facilitate integration and specialization: MaizeGDB (maize), SorghumBase (sorghum), Oryzabase (rice), and Wheat@URGI (wheat). These resources demonstrate the value of organism-specific repositories for enhancing data availability.

General public databases (NCBI, UniProt, EnsemblPlants) maintain enormous scales of biological data for numerous organisms. However, tef-related data are distributed across these resources, making them not readily available or convenient for researchers focused specifically on Eragrostis tef.

Compared with widely used crop-specific databases, TefDB focuses specifically on integrating Eragrostis tef resources into a single environment, making data access and retrieval easier for tef researchers. TefDB is a recent emerging platform developed for tef resource management and analysis, integrating various biological data and bioinformatics tools together into a single system. Table 12 presents a feature comparison of TefDB with existing biological databases.

**Table 12:** Feature comparison of TefDB with existing biological databases (NCBI, UniProt, EnsemblPlants, plantTFDB), highlighting species-specificity, integrated tools, data submission portals, and data types.

Features	TefDB	Ncbi	UniProt	Ensembl plants	plantTFDB
Tef bio-data available	yes	yes	yes	yes	yes
Species-specific for *E. te*f	Yes	No (General)	No (General)	No (General)	No (but indexes tef)
Integrated/linked analysis tools	BLAST, JBrowse, Primer3Plus, & Clustal Omega	BLAST, COBALT, CGV, Primer-BLAST, *etc*.	BLAST, Clustal Omega, ID mapping, & peptide search	BLAST,BioMart, Variant Effect Predictor, ID converter, *etc*.	BLAST, TF prediction, GO, ID mapping, *etc*.
Data Submission Portal	Yes	Yes	No (curated only)	No (imported databases)	No (curated integration only)
Data type integrated	like genome, nucleotide, gene, protein, literature, *etc*	Multiple	Protein	Multiple	Transcription factor/gene family
Data source	NCBI, UniProt, Ensembl Plants, *etc*.	INSDC partners (NCBI, EMBL-EBI, DDBJ), direct submissions, *etc*.	GenBank/EMBL translations, literature, PDB, *etc*.	ENA, NCBI, Phytozome, *etc*.	TAIR10, MSU, UniProt, *etc*.

## Conclusion and recommendations

### Conclusion

The TefDB platform was successfully developed as a web-based database for *Eragrostis tef*, integrating diverse biological datasets, literature, and bioinformatics tools, including BLAST, JBrowse 2, Clustal Omega, and Primer3Plus. The platform enables comprehensive data retrieval, visualization, sequence analysis, and primer design within a single environment, thereby streamlining workflows for tef researchers.

Evaluation of the platform demonstrates that it meets functional requirements, provides an intuitive interface, maintains data integrity, and ensures accessibility. Functional, usability, and technical testing indicate that the system is stable and suitable for prototype-level research applications. The integration of bioinformatics tools allows users to transition seamlessly from data retrieval to analysis, reducing reliance on external tools and enhancing research efficiency.

Compared with general repositories such as NCBI and EnsemblPlants, TefDB offers specialized, tef-focused coverage, providing integrated datasets and analytical tools tailored to local and regional research needs. This specificity enhances its practical utility while complementing existing general-purpose plant genomic databases.

Despite its achievements, TefDB has limitations. These include a preliminary data scope, reliance on externally linked tools, and the absence of HTTPS encryption.

### Recommendations

Based on the development and evaluation of TefDB, the following recommendations are proposed:

**Scalability improvements**: Optimize database performance to efficiently handle larger datasets.

**Enhanced security**: Implement advanced authentication methods, user roles, and data protection mechanisms.

**User feedback integration**: Conduct formal usability studies with multiple researchers to refine the interface and functionality.

**Tool integration expansion**: Consider integrating additional bioinformatics tools or automated pipelines for more complex workflows.

**Regular data updates and incorporating local varieties' data**: Establish procedures to maintain dataset accuracy and relevance over time, and integrating the local varieties data including, snp and vcf.

**Implement HTTPS and move JWT authentication to production**. This will encrypt all traffic between clients and the server, making the existing JWT authentication and CSRF protection effective. This is the highest-priority recommendation before the platform can be used for sensitive data.

**Track and report versioning and access** dates for all integrated public data.

Finally, one poignant recommendation is that the expertise and experience gained in developing TefDB is transferable to including platforms for other orphan crops such as finger millet, *enset*, etc. It would be of distinct advantage if such platforms are developed and accessed through a knowledge-base portal dedicated to orphans crops.

## Supplementary Material

baag051_Supplemental_File

## Data Availability

All datasets integrated into TefDB are publicly available from NCBI (https://www.ncbi.nlm.nih.gov), UniProt (https://www.uniprot.org), and EnsemblPlants (https://plants.ensembl.org). The source code for the TefDB platform is openly accessible at https://github.com/bioinfo33/MyProject. No new datasets were generated during this study.
