# Supplementary material for: Developing a web-based platform for Eragrostis tef resource management and analysis
Source: Database (Oxford). 2026 Aug 25;2026:baag051. doi: 10.1093/database/baag051 (PMC13504265; doi:10.1093/database/baag051)
Supplement: Supplementary file 1 [file baag051_supplemental_file.docx]

**Supplementary Data**

# **APPENDICES**

**Appendix A: Survey Questionnaires for Requirements Analysis**

Bioinformatics Platform of E.tef Data Management and Analysis

**Section 1: Personal particulars**

This section collects basic information such as age, gender, and occupation to help categorize responses. All details will be kept confidential.

1. Name (full name ): _________________

2. Email: __________________

3. Gender: ☐ Male	☐ Female

4. Age group: ☐ under 31	☐ 31-55	☐ above 55

5. Educational level: ☐ diploma ☐ B.Sc. ☐ M.Sc. ☐ PhD

6. Role: ☐ Student ☐ researcher ☐ educators/teachers ☐ bioinformatician ☐ Postdoctoral researcher ☐ Others (please specify): ___________________________________________________)

7. Institution/Organization: ________________________________

8. Country:_________________________________

**Section 2: Expertise and research focus**

This section helps to understand your experience and areas of interest related to bioinformatics and Eragrostis tef. Your response will guide in defining the scope of data and functionality of the proposed platform

9. What best describes your field of research or educational endeavor?

☐ Agriculture/Agronomy ☐ Bioinformatics/Computational Biology ☐ Biotechnology ☐ Data Science/Computer Science ☐ Plant Biology ☐ Others (please specify): __________________________________________)

10. What describe(s) the nature of your work or research interest (select all that apply)?

☐ Desk-based / In silico ☐ Field/Farm ☐ Programming/Algorithm ☐ Wet laboratory ☐ Others (please specify): __________________________________________)

11. What is your experience level in your primary field of work or research?

☐ begginer ☐ intermediate ☐ advanced ☐ expert

12. List up-to three plant species you most often work on or are interested in (except E. tef)? (eg: Zea mays, Oryza sativa, ..., etc)

13. What biological data do you work with? (Select all that apply)

☐ Gene regulation ☐ Genomic/gene sequence ☐ Metabolomic/bioloigcal network ☐ Proteomic/protein sequence ☐ Phenotypic ☐ Transcriptomic ☐ Other (please specify): ______________________________________)

**Section 3: Bioinformatic Tools and Platform**

14. How familiar are you with biological databases? ☐ very familiar ☐ some familiar ☐ not familiar

15. What bioinformatic tools and platform are you familiar with?

16. What specialized or advanced bioinformatic tools do you use in your research?

17. Which bioinformtic tools do you routinely use or know about (tick all that apply|)
☐ Sequence alignment (e.g., BLAST) ☐ Expression analysis (e.g., DESeq2, edgeR) ☐ Functional annotation (e.g., InterPro, GO) ☐ Genome browsers (e.g., Ensembl, UCSC) ☐ Other (please specify): ___________________________________________)

18. . What general purpose databases do you use frequently (tick all that apply)?
☐ EBI/EMBL ☐ Ensembl ☐ Gramene ☐ NCBI ☐ TAIR ☐ UniProt ☐ Other (please specify): ____________________________)

19. What one specialized species/genus specific database have used or are using most often (eg. MaizeGDB, GrainGenes, SorghumBase, etc.) provide the URL link.?

**Section 4: Platform Expectations & Feedback**

In this section, we invite you to share your expectations, needs, and ideas for developing and/or improving a bioinformatics platform dedicated to E. tef. Your feedback is essential to ensure the platform is user-friendly, relevant, and scientifically valuable.

20. How useful do you think it will be to have a database and allied bioinformtic tools specifically for E. tef ?

☐ Extremely useful ☐ Very useful ☐ Useful ☐ Somewhat useful ☐ Not useful

If you choose 'Not useful', please tell us why: ______________________________________)

21. What types of data would you like to access from the proposed E. tef platform, and in which formats? (Example: Gene sequences (fasta), Genome annotation data (GFF3), Expression profiles (csv), Protein data (JSON), etc.)

22. What types of interactivity would you like the platform to support for accessing and working with data? (Select all that apply) ☐ Download data in preferred formats ☐ Upload your own datasets for analysis or comparison ☐ Search and filter datasets using custom criteria ☐ Visualize data (e.g., genome browser, phylogenetic trees) ☐ Run basic bioinformatics analyses (e.g., alignments, annotation, BLAST) ☐ Customize or export analysis workflows ☐Share data or results with collaborators, ☐ Generate reports or export summaries ☐ Other (please specify): ___________)

23. What existing popular bioinformatic analytical tools (e.g., BLAST, CLUSTAL Omega, Burrows-Wheeler Aligner, SPAdes, etc.) would you like to see integrated in the proposed E. tef database?

24. What is your most preferred access device?

25. How important is a user-friendly interface to you? ☐ very important ☐ important ☐ slightly important ☐ not important

26. Do you think the E. tef database should be bilingual (English and Amharic)? (Yes, or No)

27. How frequently would you use this E. tef dedicated bioinformatic platform when it is made available? ☐ very frequetly ☐ frequently ☐ regular but infrequently ☐ very infrequently

28. What challenges do you currently face in using existing platforms in accessing and analyzing E. tef data?

29. What are your expectations from this platform to help you with your work?

30. Please let us know if you have additional suggestions or recommendations (features, functionalities, aesthetics, etc.) that will help in ensuring that the scientific community gains maximum benefit from the proposed E. tef database?

31. Finally, we would like to name the E. tef database with an appropriate name that is catchy and appropriate. Which of the following proposed names do you prefer? Or, do you have another name in mind?

Appendix A.1: Result of requirement analysis survey from respondents

**Appendix B: Screenshots of the TefDB Platform**

The following figures demonstrate the system interface and integrated bioinformatics tools.

Figure B.1: Home Page of TefDB

**Figure B.2 Log in page**

**Figure B.3 Sign up page**

**Figure B.4 Feedback form**

Figure B.5 **Data submission form**

Figure B.6 **JBrowse Page**

Figure B.7 **BLAST work Page**

Figure B.8 BLAST result **Page**

Figure B.9 Functional API Testing Results Using Postman

Figure B.9 Performance API Testing Results Using Postman

Figure B.10 Functional API Testing Results for Recent-Activity Using Postman

Figure B.11 Functional API Testing Results for Dataset-submission Using Postman

Figure B.12 Functional API Testing Results for Feedback Using Postman

Figure B.13 Functional API Testing Results for Login Using Postman for both successful and error check

Figure B.14 Functional API Testing Results for Register Using Postman

for both successful and error check

Figure B.15 Results of User interface testing by Selenium tools

This shows the count of detail data, invalid input , and status with Pass and fail (for invalid input)

**Appendix C: Some Backend components**

Figure C.1 Some Models of the Entity

Figure C.2 Admin

Figure C.3 View

Figure C.4 Serializers

Figure C.5 URL

Appendix D: Component code for front-end and tables in database

Figure D.1 Nucleotide component screenshot

Figure D.2 Gene component screenshot

Figure D.3 Nucleotide table screenshot from databases

Figure D.4 PubMed table screenshot from databases

Figure D.5 Gene table screenshot from databases

Appendix E: Sample of API Responses

Example of a RESTful API JSON response queries for Taxonomy and Nucleotide tables.

Figure E.1 **Taxonomy API response**

Taxonomy

```
GET /api/taxonomy/
HTTP 200 OK
Allow: GET, HEAD, OPTIONS
Content-Type: application/json
Vary: Accept

{
    "count": 1,
    "next": null,
    "previous": null,
    "results": [
        {
            "tax_id": 110835,
            "scientific_name": "Eragrostis tef",
            "synonyms": [
                "Poa tef"
            ],
            "common_names": [
                "tef"
            ],
            "authority_names": [
                "Eragrostis tef (Zuccagni) Trotter, 1918",
                "Poa tef Zuccagni, 1775"
            ],
            "rank": "species",
            "parent_tax_id": 38413,
            "division": "Plants and Fungi",
            "genetic_code_name": "Standard",
            "genetic_code_id": 1,
            "mito_genetic_code_name": "Standard",
            "mito_genetic_code_id": 1,
            "lineage": "cellular organisms; Eukaryota; Viridiplantae; Streptophyta; Streptophytina; Embryophyta; Tracheophyta; Euphyllophyta; Spermatophyta; Magnoliopsida; Mesangiospermae; Liliopsida; Petrosaviidae; commelinids; Poales; Poaceae; PACMAD clade; Chloridoideae; Eragrostideae; Eragrostidinae; Eragrostis",
            "lineage_json": [
                {
                    "Rank": "cellular root",
                    "TaxId": "131567",
                    "ScientificName": "cellular organisms"
                },
                {
                    "Rank": "domain",
                    "TaxId": "2759",
                    "ScientificName": "Eukaryota"
                },
                {
                    "Rank": "kingdom",
                    "TaxId": "33090",
                    "ScientificName": "Viridiplantae"
                },
                {
                    "Rank": "phylum",
                    "TaxId": "35493",
                    "ScientificName": "Streptophyta"
                },
                {
                    "Rank": "subphylum",
                    "TaxId": "131221",
                    "ScientificName": "Streptophytina"
                },
                {
                    "Rank": "clade",
                    "TaxId": "3193",
                    "ScientificName": "Embryophyta"
                },
                {
                    "Rank": "clade",
                    "TaxId": "58023",
                    "ScientificName": "Tracheophyta"
                },
                {
                    "Rank": "clade",
                    "TaxId": "78536",
                    "ScientificName": "Euphyllophyta"
                },
                {
                    "Rank": "clade",
                    "TaxId": "58024",
                    "ScientificName": "Spermatophyta"
                },
                {
                    "Rank": "class",
                    "TaxId": "3398",
                    "ScientificName": "Magnoliopsida"
                },
                {
                    "Rank": "clade",
                    "TaxId": "1437183",
                    "ScientificName": "Mesangiospermae"
                },
                {
                    "Rank": "clade",
                    "TaxId": "4447",
                    "ScientificName": "Liliopsida"
                },
                {
                    "Rank": "subclass",
                    "TaxId": "1437197",
                    "ScientificName": "Petrosaviidae"
                },
                {
                    "Rank": "clade",
                    "TaxId": "4734",
                    "ScientificName": "commelinids"
                },
                {
                    "Rank": "order",
                    "TaxId": "38820",
                    "ScientificName": "Poales"
                },
                {
                    "Rank": "family",
                    "TaxId": "4479",
                    "ScientificName": "Poaceae"
                },
                {
                    "Rank": "clade",
                    "TaxId": "147370",
                    "ScientificName": "PACMAD clade"
                },
                {
                    "Rank": "subfamily",
                    "TaxId": "147371",
                    "ScientificName": "Chloridoideae"
                },
                {
                    "Rank": "tribe",
                    "TaxId": "147436",
                    "ScientificName": "Eragrostideae"
                },
                {
                    "Rank": "subtribe",
                    "TaxId": "751752",
                    "ScientificName": "Eragrostidinae"
                },
                {
                    "Rank": "genus",
                    "TaxId": "38413",
                    "ScientificName": "Eragrostis"
                }
            ],
            "properties": {
                "pgcode": 11
            },
            "create_date": "1999-12-16T17:07:00Z",
            "update_date": "2017-06-14T15:56:24Z",
            "pub_date": "2001-12-20T00:01:00Z"
        }
    ]
}
```

Figure E.2 **Nucleotide API response**

```
GET /api/nucleotide/
HTTP 200 OK
Allow: GET, HEAD, OPTIONS
Content-Type: application/json
Vary: Accept

{
    "count": 3681,
```

"next": "[http://197.156.76.122/api/nucleotide/?page=2](http://197.156.76.122/api/nucleotide/?page=2)",

```
    "previous": null,
    "results": [
        {
            "id": 3988,
            "fasta": "/media/nucleotides/3988.fasta",
            "accession": "AJ279199",
            "version": "1",
            "definition": "Eragrostis tef partial 18S rRNA gene",
            "title": "Eragrostis tef partial 18S rRNA gene",
            "organism": "Eragrostis tef",
            "source": "Eragrostis tef",
            "authors": "Espelund,M., Bekele,E., Holst-Jensen,A., Jakobsen,K.S., Nordal,I., Holst-Jensen,A.",
            "cultivar": null,
            "clone": null,
            "dev_stage": null,
            "lab_host": null,
            "note": null,
            "gi": 17906973,
            "sequence_type": "DNA",
            "mol_type": "DNA",
            "sequence_length": 449,
            "sequence": "cgacccttctgccggcgatgcgctcctggccttaattggccgggtcgtgcctccggcgctgttactttgaagaaattagagtgctcaaagcaagccatcgctctggatacattagcatgggataacatcataggattccggtcctattgtgttggcttcgggatcggagtaatgattaatagggacagtcgggggcattcgtatttcatagtcagaggtgaaattcttggatttatgaaagacgaacaactgcgaaagcatttgccaaggatgttttcattaatcaagaacgaaagttgggggctcgaagacgatcagataccgtcctagtctcaaccataaacgatgccgaccagggatcggcggatgttgcttataggactccgccggcaccttatgagaaatcaaagtctttgggttccggggggagtatggtcgcaaggctgaaa",
            "genbank": "LOCUS       AJ279199                 449 bp    DNA     linear   PLN 06-OCT-2004\nDEFINITION  Eragrostis tef partial 18S rRNA gene.\nACCESSION   AJ279199\nVERSION     AJ279199.1\nKEYWORDS    18S ribosomal RNA; 18S rRNA gene.\nSOURCE      Eragrostis tef\n  ORGANISM  Eragrostis tef\n            Eukaryota; Viridiplantae; Streptophyta; Embryophyta; Tracheophyta;\n            Spermatophyta; Magnoliopsida; Liliopsida; Poales; Poaceae; PACMAD\n            clade; Chloridoideae; Eragrostideae; Eragrostidinae; Eragrostis.\nREFERENCE   1\n  AUTHORS   Espelund,M., Bekele,E., Holst-Jensen,A., Jakobsen,K.S. and\n            Nordal,I.\n  TITLE     A molecular genetic analysis of Eragrostis tef (Zucc.) Trotter:\n            non-coding regions of chloroplast DNA, 18S rDNA and the\n            transcription factor VP1\n  JOURNAL   Hereditas 132 (3), 193-202 (2000)\n   PUBMED   11075514\nREFERENCE   2  (bases 1 to 449)\n  AUTHORS   Holst-Jensen,A.\n  TITLE     Direct Submission\n  JOURNAL   Submitted (15-DEC-1999) Holst-Jensen A., Section of Feed and Food\n            Microbiology, National Veterinary Institute, POBox 8156 Dep., 0033\n            Oslo, NORWAY\nFEATURES             Location/Qualifiers\n     source          1..449\n                     /organism=\"Eragrostis tef\"\n                     /mol_type=\"genomic DNA\"\n                     /db_xref=\"taxon:110835\"\n     gene            <1..>449\n                     /gene=\"18S rRNA\"\n     rRNA            <1..>449\n                     /gene=\"18S rRNA\"\n                     /product=\"18S ribosomal RNA\"\nORIGIN      \n        1 cgacccttct gccggcgatg cgctcctggc cttaattggc cgggtcgtgc ctccggcgct\n       61 gttactttga agaaattaga gtgctcaaag caagccatcg ctctggatac attagcatgg\n      121 gataacatca taggattccg gtcctattgt gttggcttcg ggatcggagt aatgattaat\n      181 agggacagtc gggggcattc gtatttcata gtcagaggtg aaattcttgg atttatgaaa\n      241 gacgaacaac tgcgaaagca tttgccaagg atgttttcat taatcaagaa cgaaagttgg\n      301 gggctcgaag acgatcagat accgtcctag tctcaaccat aaacgatgcc gaccagggat\n      361 cggcggatgt tgcttatagg actccgccgg caccttatga gaaatcaaag tctttgggtt\n      421 ccggggggag tatggtcgca aggctgaaa\n//\n\n",
            "asn1": "Seq-entry ::= seq {\n  id {\n    embl {\n      accession \"AJ279199\",\n      version 1\n    },\n    gi 17906973\n  },\n  descr {\n    title \"Eragrostis tef partial 18S rRNA gene\",\n    source {\n      org {\n        taxname \"Eragrostis tef\",\n        db {\n          {\n            db \"taxon\",\n            tag id 110835\n          }\n        },\n        orgname {\n          name binomial {\n            genus \"Eragrostis\",\n            species \"tef\"\n          },\n          attrib \"specified\",\n          lineage \"Eukaryota; Viridiplantae; Streptophyta; Embryophyta;\n Tracheophyta; Spermatophyta; Magnoliopsida; Liliopsida; Poales; Poaceae;\n PACMAD clade; Chloridoideae; Eragrostideae; Eragrostidinae; Eragrostis\",\n          gcode 1,\n          mgcode 1,\n          div \"PLN\",\n          pgcode 11\n        }\n      }\n    },\n    molinfo {\n      biomol genomic\n    },\n    pub {\n      pub {\n        sub {\n          authors {\n            names std {\n              {\n                name name {\n                  last \"Holst-Jensen\",\n                  initials \"A.\"\n                }\n              }\n            },\n            affil str \"Holst-Jensen A., Section of Feed and Food Microbiology,\n National Veterinary Institute, POBox 8156 Dep., 0033 Oslo, NORWAY\"\n          },\n          medium other,\n          date std {\n            year 1999,\n            month 12,\n            day 15\n          }\n        }\n      }\n    },\n    pub {\n      pub {\n        pmid 11075514,\n        article {\n          title {\n            name \"A molecular genetic analysis of Eragrostis tef (Zucc.)\n Trotter: non-coding regions of chloroplast DNA, 18S rDNA and the\n transcription factor VP1.\"\n          },\n          authors {\n            names std {\n              {\n                name name {\n                  last \"Espelund\",\n                  initials \"M.\"\n                }\n              },\n              {\n                name name {\n                  last \"Bekele\",\n                  initials \"E.\"\n                }\n              },\n              {\n                name name {\n                  last \"Holst-Jensen\",\n                  initials \"A.\"\n                }\n              },\n              {\n                name name {\n                  last \"Jakobsen\",\n                  initials \"K.S.\"\n                }\n              },\n              {\n                name name {\n                  last \"Nordal\",\n                  initials \"I.\"\n                }\n              }\n            },\n            affil str \"Division of Molecular Biology, University of Oslo,\n Norway.\"\n          },\n          from journal {\n            title {\n              iso-jta \"Hereditas\",\n              ml-jta \"Hereditas\",\n              issn \"0018-0661\",\n              name \"Hereditas\"\n            },\n            imp {\n              date std {\n                year 2000\n              },\n              volume \"132\",\n              issue \"3\",\n              pages \"193-202\",\n              language \"eng\",\n              pubstatus ppublish,\n              history {\n                {\n                  pubstatus pubmed,\n                  date std {\n                    year 2000,\n                    month 11,\n                    day 15,\n                    hour 11,\n                    minute 0\n                  }\n                },\n                {\n                  pubstatus medline,\n                  date std {\n                    year 2001,\n                    month 2,\n                    day 28,\n                    hour 10,\n                    minute 1\n                  }\n                },\n                {\n                  pubstatus other,\n                  date std {\n                    year 2000,\n                    month 11,\n                    day 15,\n                    hour 11,\n                    minute 0\n                  }\n                }\n              }\n            }\n          },\n          ids {\n            doi \"10.1111/j.1601-5223.2000.00193.x\",\n            pubmed 11075514\n          }\n        }\n      },\n      reftype no-target\n    },\n    embl {\n      div pln,\n      creation-date std {\n        year 2001,\n        month 12,\n        day 15\n      },\n      update-date std {\n        year 2004,\n        month 10,\n        day 6\n      },\n      keywords {\n        \"18S ribosomal RNA\",\n        \"18S rRNA gene\"\n      },\n      xref {\n        {\n          dbname name \"SILVA-SSU\",\n          id {\n            str \"AJ279199\"\n          }\n        }\n      }\n    },\n    create-date std {\n      year 2001,\n      month 12,\n      day 15\n    },\n    update-date std {\n      year 2004,\n      month 10,\n      day 6\n    }\n  },\n  inst {\n    repr raw,\n    mol dna,\n    length 449,\n    seq-data ncbi2na '615F79698E675E97C3E96ADB975A67BC7F8203C8B9D024253677A313\nC93A8C134CA3D6B5CFBBE9F6A368B0E3C32A12DAA93DB3F4CB48AE03DFA3F38086041E6024FE50\nA3BFD3C3420602FAA9D8218D2316D72DD053018E5852A369A3BE7CCA1D65A45F3880D02DFEAF5A\nAA2CEB6429E000'H\n  },\n  annot {\n    {\n      data ftable {\n        {\n          data rna {\n            type rRNA,\n            ext name \"18S ribosomal RNA\"\n          },\n          partial TRUE,\n          location int {\n            from 0,\n            to 448,\n            id gi 17906973,\n            fuzz-from lim lt,\n            fuzz-to lim gt\n          }\n        },\n        {\n          data gene {\n            locus \"18S rRNA\"\n          },\n          partial TRUE,\n          location int {\n            from 0,\n            to 448,\n            id gi 17906973,\n            fuzz-from lim lt,\n            fuzz-to lim gt\n          }\n        }\n      }\n    }\n  }\n}\n\n",
            "gff3": "LOCUS       AJ279199                 449 bp    DNA     linear   PLN 06-OCT-2004\nDEFINITION  Eragrostis tef partial 18S rRNA gene.\nACCESSION   AJ279199\nVERSION     AJ279199.1\nKEYWORDS    18S ribosomal RNA; 18S rRNA gene.\nSOURCE      Eragrostis tef\n  ORGANISM  Eragrostis tef\n            Eukaryota; Viridiplantae; Streptophyta; Embryophyta; Tracheophyta;\n            Spermatophyta; Magnoliopsida; Liliopsida; Poales; Poaceae; PACMAD\n            clade; Chloridoideae; Eragrostideae; Eragrostidinae; Eragrostis.\nREFERENCE   1\n  AUTHORS   Espelund,M., Bekele,E., Holst-Jensen,A., Jakobsen,K.S. and\n            Nordal,I.\n  TITLE     A molecular genetic analysis of Eragrostis tef (Zucc.) Trotter:\n            non-coding regions of chloroplast DNA, 18S rDNA and the\n            transcription factor VP1\n  JOURNAL   Hereditas 132 (3), 193-202 (2000)\n   PUBMED   11075514\nREFERENCE   2  (bases 1 to 449)\n  AUTHORS   Holst-Jensen,A.\n  TITLE     Direct Submission\n  JOURNAL   Submitted (15-DEC-1999) Holst-Jensen A., Section of Feed and Food\n            Microbiology, National Veterinary Institute, POBox 8156 Dep., 0033\n            Oslo, NORWAY\nFEATURES             Location/Qualifiers\n     source          1..449\n                     /organism=\"Eragrostis tef\"\n                     /mol_type=\"genomic DNA\"\n                     /db_xref=\"taxon:110835\"\n     gene            <1..>449\n                     /gene=\"18S rRNA\"\n     rRNA            <1..>449\n                     /gene=\"18S rRNA\"\n                     /product=\"18S ribosomal RNA\"\nORIGIN      \n        1 cgacccttct gccggcgatg cgctcctggc cttaattggc cgggtcgtgc ctccggcgct\n       61 gttactttga agaaattaga gtgctcaaag caagccatcg ctctggatac attagcatgg\n      121 gataacatca taggattccg gtcctattgt gttggcttcg ggatcggagt aatgattaat\n      181 agggacagtc gggggcattc gtatttcata gtcagaggtg aaattcttgg atttatgaaa\n      241 gacgaacaac tgcgaaagca tttgccaagg atgttttcat taatcaagaa cgaaagttgg\n      301 gggctcgaag acgatcagat accgtcctag tctcaaccat aaacgatgcc gaccagggat\n      361 cggcggatgt tgcttatagg actccgccgg caccttatga gaaatcaaag tctttgggtt\n      421 ccggggggag tatggtcgca aggctgaaa\n//\n\n",
            "created_at": "2025-10-19T14:03:46.673171Z",
            "updated_at": "2026-02-16T13:53:56.581822Z",
            "tax_id": 110835,
            "biosample_id": null,
            "pubmed_id": 11075514
        },
```

**Appendix F: TefDB Platform User Feedback & Validation Survey**

A comprehensive database (knowledge-base) for tef was developed called TefDB and is now under evaluation. Thank you for your interest in participating in evaluating the TefDB. Your feedback and input on its usefulness, usability and effectiveness will help us further refine it.

The database can be found on http://197.156.76.122/home. Please visit the site and provide your response in this survey to validate the system, and guide in its future improvement.

The survey will take 5 - 6 minutes, and your responses and personal identifiers will be kept in strict confidence.

Thank you.

**Section A: User Background**

1. What best describes your role?

☐ Student

☐ Researcher

☐ Academic staff

☐ Other (please specify): ___________

2. Which best describes your primary area of discipline?

☐ Agronomy

☐ Molecular biology

☐ Biotechnology

☐ Bioinformatics

☐ Others (please indicate) _____________

3. What is your experience level in your primary field?

☐ Beginner

☐ Intermediate

☐ Advanced

☐ Expert

4. Do you work with Eragrostis tef species?

☐ Yes, regularly

☐ Occasionally

☐ No, but I am interested

5. How familiar are you with biological databases and bioinformatic tools?

☐ very familiar

☐ Moderately familiar

☐ Slightly familiar

☐ Not familiar

**Section B: Platform Content & Data**

6. Which types of data did you access on TefDB? (Select all that apply)

☐ Genome sequences

☐ Gene

☐ Protein data

☐ Expression data

☐ Literatures

☐ SRA

☐ BioSample

☐ BioProject

☐ Other (list all): ___________

7. Are the available data types adequate for your work?

☐ Yes

☐ Partially

☐ No

☐ Not sure

8. If you answered 'No' in above question #7. What other datasets do you suggest should be included?

9. How would you rate the accuracy and relevance of the data provided?

☐ Very poor

☐ Poor

☐ Good

☐ Very good

☐ Excellent

**Section C: Tools, Features & Usability**

10. Which platform features did you use? (Select all that apply)

☐ Search, filter, and browse functions

☐ Sequence similarity search (e.g., BLAST)

☐ Data download and copy

☐ Visualization

☐ External tool integration

☐ Primer designing (e.g., Primer3Plus)

☐ Other (specify them): ___________

11. How easy was it to navigate and use the TefDB interface?

☐ Very difficult

☐ Difficult

☐ Neutral

☐ Easy

☐ Very easy

12. How satisfied are you with the performance and responsiveness of the platform?

☐ Very dissatisfied

☐ Dissatisfied

☐ Neutral

☐ Satisfied

☐ Very satisfied

13. How friendly is the user interface?

☐ Not important

☐ Slightly important

☐ Moderately important

☐ Very important

☐ Essential

**Section D: Overall Evaluation**

14. How useful is TefDB as a dedicated database for Eragrostis tef research?

☐ Not useful

☐ Slightly useful

☐ Moderately useful

☐ Very useful

☐ Extremely useful

15. How frequently do you expect to use TefDB in the future?

☐ Rarely

☐ Occasionally

☐ Monthly

☐ Weekly

☐ Very frequently

16. How satisfied are you with the overall performance and responsiveness of the platform?

☐ Very satisfied

☐ Satisfied

☐ Neutral

☐ Dissatisfied

☐ Very dissatisfied

17. What challenges, if any, did you encounter while using the platform?

_______________________________________________________________________

18. What additional features or improvements would you recommend for TefDB?

_________________________________________________________________________

**[Appendix G: TefDB Platform User Feedback & Validation Survey Results](./Appendix%20G:%20TefDB%20Platform%20User%20Feedback%20&%20Validation%20Survey%20Results)**
